# Evolutionary Dynamics and Dissemination Pattern of the SARS-CoV-2 Lineage B.1.1.33 During the Early Pandemic Phase in Brazil

**DOI:** 10.3389/fmicb.2020.615280

**Published:** 2021-02-17

**Authors:** Paola Cristina Resende, Edson Delatorre, Tiago Gräf, Daiana Mir, Fernando Couto Motta, Luciana Reis Appolinario, Anna Carolina Dias da Paixão, Ana Carolina da Fonseca Mendonça, Maria Ogrzewalska, Braulia Caetano, Gabriel Luz Wallau, Cássia Docena, Mirleide Cordeiro dos Santos, Jessylene de Almeida Ferreira, Edivaldo Costa Sousa Junior, Sandro Patroca da Silva, Sandra Bianchini Fernandes, Lucas Alves Vianna, Larissa da Costa Souza, Jean F. G. Ferro, Vanessa B. Nardy, Cliomar A. Santos, Irina Riediger, Maria do Carmo Debur, Júlio Croda, Wanderson K. Oliveira, André Abreu, Gonzalo Bello, Marilda M. Siqueira

**Affiliations:** ^1^Laboratory of Respiratory Viruses and Measles, Oswaldo Cruz Institute (IOC), Oswaldo Cruz Foundation (FIOCRUZ), SARS-CoV-2 National Reference Laboratory for the Brazilian Ministry of Health (MoH) and Regional Reference Laboratory in Americas for the Pan-American Health Organization (PAHO/WHO), Rio de Janeiro, Brazil; ^2^Departamento de Biologia, Centro de Ciencias Exatas, Naturais e da Saude, Universidade Federal do Espirito Santo, Alegre, Brazil; ^3^Instituto Gonçalo Moniz, Fundação Oswaldo Cruz, Salvador, Brazil; ^4^Unidad de Genomica y Bioinformatica, Centro Universitario Regional del Litoral Norte, Universidad de la Republica, Salto, Uruguay; ^5^Instituto Aggeu Magalhaes, Fundação Oswaldo Cruz, Recife, Brazil; ^6^Instituto Evandro Chagas, Belem, Para; ^7^Laboratorio Central de Saude Publica do Estado de Santa Catarina (LACEN-SC), Florianopolis, Brazil; ^8^Laboratorio Central de Saude Publica do Estado Espirito Santo (LACEN-ES), Vitoria, Brazil; ^9^Laboratorio Central de Saude Publica do Distrito Federal (LACEN-DF), Brazilia, Brazil; ^10^Laboratorio Central de Saude Publica de Alagoas (LACEN-AL), Maceio, Brazil; ^11^Laboratorio Central de Saude Publica da Bahia (LACEN-BA), Salvador, Brazil; ^12^Laboratorio Central de Saude Publica de Sergipe (LACEN-SE), Aracaju, Brazil; ^13^Laboratorio Central de Saude Publica de Parana (LACEN-PR), Curitiba, Brazil; ^14^Fiocruz Mato Grosso do Sul, Campo Grande, Brazil; ^15^Universidade Federal de Mato Grosso do Sul – UFMS, Campo Grande, Brazil; ^16^Hospital das Forças Armadas, Ministério da Defesa, Brasília, Brazil; ^17^Coordenadoria Geral de Laboratorios – Ministério da Saude, Brazilia, Brazil; ^18^Laboratorio de AIDS e Imunologia Molecular, Instituto Oswaldo Cruz, Fiocruz, Rio de Janeiro, Brazil

**Keywords:** coronavirus disease 2019, severe acute respiratory syndrome coronavirus-2, coronavirus, Brazil, genetic lineages, community transmission

## Abstract

A previous study demonstrates that most of severe acute respiratory syndrome coronavirus-2 (SARS-CoV-2) Brazilian strains fell in three local clades that were introduced from Europe around late February 2020. Here we investigated in more detail the origin of the major and most widely disseminated SARS-CoV-2 Brazilian lineage B.1.1.33. We recovered 190 whole viral genomes collected from 13 Brazilian states from February 29 to April 31, 2020 and combined them with other B.1.1 genomes collected globally. Our genomic survey confirms that lineage B.1.1.33 is responsible for a variable fraction of the community viral transmissions in Brazilian states, ranging from 2% of all SARS-CoV-2 genomes from Pernambuco to 80% of those from Rio de Janeiro. We detected a moderate prevalence (5–18%) of lineage B.1.1.33 in some South American countries and a very low prevalence (<1%) in North America, Europe, and Oceania. Our study reveals that lineage B.1.1.33 evolved from an ancestral clade, here designated B.1.1.33-like, that carries one of the two B.1.1.33 synapomorphic mutations. The B.1.1.33-like lineage may have been introduced from Europe or arose in Brazil in early February 2020 and a few weeks later gave origin to the lineage B.1.1.33. These SARS-CoV-2 lineages probably circulated during February 2020 and reached all Brazilian regions and multiple countries around the world by mid-March, before the implementation of air travel restrictions in Brazil. Our phylodynamic analysis also indicates that public health interventions were partially effective to control the expansion of lineage B.1.1.33 in Rio de Janeiro because its median effective reproductive number (*R*_*e*_) was drastically reduced by about 66% during March 2020, but failed to bring it to below one. Continuous genomic surveillance of lineage B.1.1.33 might provide valuable information about epidemic dynamics and the effectiveness of public health interventions in some Brazilian states.

## Introduction

Coronavirus disease 2019 (COVID-19), the disease caused by severe acute respiratory syndrome coronavirus-2 (SARS-CoV-2), has gone pandemic, leading to high rates of acute respiratory syndrome, hospitalization, and death worldwide (Zhu et al., 2020). Brazil, the third most affected country in the world so far, has reported over 5.7 million cases and over 163,000 deaths (last update November 12, 2020) (Dong et al., 2020). The first positive case of SARS-CoV-2 infection in Brazil was reported on February 25, 2020 in an individual traveling from Europe to Sao Paulo metropolitan region (de Souza et al., 2020), and during the following 2 weeks, the virus was detected in all country regions (Jesus et al., 2020).

The rapid worldwide genomic surveillance of SARS-CoV-2, mainly shared via the EpiCoV database in the GISAID initiative^1^ coupled with the development of real-time interactive platforms for pathogen genomic data^2^, has been crucial for managing this public health emergency, enabling the tracking of viral transmission patterns as the epidemic unfolds. According to the nomenclature systems for designation of SARS-CoV-2 lineages^3^, there are currently two major groups, designated as lineages A and B, that have diversified while the virus spread across the world, in several phylogenetic subclades characterized by the presence of particular sets of shared mutations (Shu and McCauley, 2017; Hadfield et al., 2018; Rambaut et al., 2020). The nomenclature implemented in the Pangolin software defined further SARS-CoV-2 active lineages by assigning a numerical value to descendants from either lineage A or B that fulfill a set of conditions. A maximum of three sublevels is allowed (e.g., A.1.1.1); after that, new descendent lineages are given a letter. The SARS-CoV-2 lineage B.1 was initially identified as the most common variant in Europe and is currently also the predominant viral lineage in the Americas (Candido et al., 2020; Deng et al., 2020; Fauver et al., 2020; Franco et al., 2020; Gonzalez-Reiche et al., 2020; Laiton-Donato et al., 2020; Paiva et al., 2020; Paniz-Mondolfi et al., 2020; Ramirez et al., 2020; Salazar et al., 2020; Taboada et al., 2020; Xavier et al., 2020).

Genomic epidemiology has been a useful tool to track the community transmission of SARS-CoV-2, revealing that its epidemics in Oceania (Rockett et al., 2020; Seemann et al., 2020), Europe (Bluhm et al., 2020; Dellicour et al., 2020; Díez-Fuertes et al., 2020; Gambaro et al., 2020; Gudbjartsson et al., 2020; Miller et al., 2020; Oude Munnink et al., 2020), and the Americas (Deng et al., 2020; Gonzalez-Reiche et al., 2020; Worobey et al., 2020) resulted from multiple independent introductions followed by local dissemination of some viral strains. One previous study estimated that most (76%, *n* = 370/490) of the Brazilian SARS-CoV-2 strains recovered from different country regions group into three main clades designated with numbers (Candido et al., 2020). The clade 1, characterized by one non-synonymous mutation in the spike protein (G25088T), was subsequently classified by the Pangolin nomenclature as lineage B.1.1.28, and the clade 2, defined by two non-synonymous synapomorphies in ORF6 (T27299C/I33T) and the nucleocapsid protein (T29148C/I292T), was classified as lineage B.1.1.33. According to that study, the lineage B.1.1.28 circulates predominantly in the state of Sao Paulo and its origin was dated to February 28 (February 21 to March 4), while lineage B.1.1.33 circulates in different Brazilian states, and its origin was traced to February 22 (February 17–24), 2020. The precise dispersion pattern, demographical dynamics, and global prevalence of these major Brazilian clades are currently unclear.

Here we perform an in-depth analysis of the origin and spatiotemporal dissemination dynamics of B.1.1.33 lineage in Brazil and globally. To this end, we generated 190 SARS-CoV-2 whole-genomes sampled in 13 different Brazilian states during the first 2 months of the COVID-19 epidemic. New B.1.1.33 viral sequences identified here were combined with other Brazilian and global SARS-CoV-2 genomes containing the same synapomorphic mutations and then subjected to maximum likelihood (ML) and Bayesian phylogenetic analyses.

## Materials and Methods

### Sampling and Ethical Aspects

We recovered viral whole-genomes (>99% coverage) from nasopharyngeal–throat combined swabs collected from 190 individuals with confirmed SARS-CoV-2 infection, who underwent testing and/or genomic sequencing at the Laboratory of Respiratory Viruses and Measles-Oswaldo Cruz Institute-FIOCRUZ, in Rio de Janeiro, the Technological Platform and Bioinformatic Core-Aggeu Magalhaes Institute-FIOCRUZ, in Pernambuco, and the Evandro Chagas Institute, in Para Brazil between February 29 and April 30, 2020. Samples were collected from clinically ill individuals (between the first and the 11th day after their first symptoms) or from asymptomatic individuals suspicious of SARS-CoV-2 infection that reside in 13 different Brazilian states from the Southeastern (Rio de Janeiro and Espirito Santo), Central-Western (Distrito Federal), Northern (Acre, Amapa, and Para), Northeastern (Alagoas, Bahia Maranhao, Pernambuco, and Sergipe), and Southern (Parana and Santa Catarina) regions (Supplementary Table 1). Samples were conserved in the viral transport medium at 4–8°C up to processing. This study was approved by the FIOCRUZ-IOC Ethics Committee (68118417.6.0000.5248 and CAAE 32333120.4.0000.5190) and the Brazilian Ministry of Health SISGEN (A1767C3).

### Nucleic Acid Isolation and RT–qPCR

The viral RNA was extracted manually from 140 μl of clinical samples using QIAamp Viral RNA Mini kit (QIAGEN, Hilden, Germany) or automatedly using 300 μl of the sample and Perkin-Elmer Chemagic machine/chemistry, according to the manufacturer’s instructions. SARS-CoV-2-positive cases were confirmed by real-time RT-PCR assays using the SARS-CoV-2 Molecular E/RP kit (Biomanguinhos, Rio de Janeiro, Brazil) based on the protocol previously designed by Corman et al. (2020). Amplifications were conducted in the ABI7500 platform using the following conditions: reverse transcription (50°C, 15 min), reverse transcriptase inactivation and DNA polymerase activation (95°C, 2 min), followed by 45 cycles of DNA denaturation (95°C, 20 s) and annealing-extension (58°C, 30 s). The fluorescence data was collected in the annealing-extension step, and all samples with sigmoid curves crossing the threshold line up to cycle 40 were named positive. Negative and positive controls were included in each extraction and real-time RT-PCR batch.

### SARS-CoV-2 Whole-Genome Amplification and Sequencing

Total RNA from positive samples presenting Ct values up to 300 for gene E was reverse transcribed using SuperScript^TM^ IV First Strand Synthesis System (Invitrogen). Two multiplex PCR reactions using the primer scheme previously described (Resende et al., 2020b) (Pool A = nine amplicons and Pool B = eight amplicons) were performed using the Q5^®^ High-Fidelity DNA Polymerase (NEB). Amplicons were purified using Agencourt AMPure XP beads (Beckman Coulter^TM^), and the DNA quantified with Qubit 4 Fluorometer (Invitrogen) using the Qubit dsDNA HS Assay kit (Invitrogen) and sequenced using Illumina MiSeq or NextSeq (San Diego, CA, United States) and Nanopore (Oxford, United Kingdom). Illumina short reads DNA libraries were generated from the pooled amplicons using Nextera XT DNA Sample Preparation kit (Illumina, San Diego, CA, United States) according to the manufacturer specifications. The size distribution of these libraries was evaluated using a 2100 Bioanalyzer (Agilent, Santa Clara, United States), and the samples were pair-end sequenced (Micro V2, 300 cycles) on a MiSeq/NextSeq equipment (Illumina, San Diego, United States) in around 18 h. The Nanopore library protocol is optimized for long reads (2 kb amplicons) (Resende et al., 2020b). Library preparation was conducted using Ligation Sequencing 1D (SQK-LSK109 Oxford Nanopore Technologies (ONT) and Native Barcoding kit 1–24 (ONT), according to the manufacturer’s instructions. After end repair using the NEBNext^®^ Ultra^TM^ II End Repair/dA-Tailing Module (New England Biolabs, NEB), the native barcodes were attached using a NEBNext^®^ Ultra^TM^ II Ligation Module (NEB). Up to 23 samples were pooled for sequencing in the same flow cell (FLOMIN106 flow cell R9.4.1), and a negative mock sample was loaded in each run for validation. The sequencing was performed for 12 h using the high-accuracy base calling in the MinKNOW software; however, the run was monitored by RAMPART^4^ allowing us to stop the assay after 2 h, when ≥20 × depth for all amplicons was achieved.

### Data Analysis to Recover the SARS-CoV-2 Whole-Genome Consensus Sequences

Demultiplexed fastq files generated by Illumina sequencing were used as the input for the analysis. Reads were trimmed based on quality scores with a cutoff of Q30, in order to remove low-quality regions, and adapter sequences were filtered. Following standard pre-processing steps, reads were mapped to the hCoV-19/Wuhan/Hu-1/2019 strain (GISAID accession number EPI_ISL_402125). Duplicate reads were removed from the alignment and the consensus sequence called at a threshold of 10×. The entire workflow was carried out in CLC Genomics Workbench software version 20.0^5^. For the ONT sequencing data, the pipeline used was an adaptation of the nCoV-2019 novel coronavirus bioinformatics protocol developed by Artic^6^. We used an earlier version of the workflow, which used Porechop^7^ to demultiplex the reads and trim off the adaptors. The mapping to the hCoV-19/Wuhan/Hu-1/2019 reference sequence was done using Minimap2 (Li, 2018) and Medaka^8^. This was all carried out within the artic-ncov2019 conda environment^9^. All genomes produced in this study can be accessed in https://www.gisaid.org/ under the accession number listed in Supplementary Table 1.

### Maximum Likelihood Phylogenetic Analyses

New Brazilian SARS-CoV-2 genome sequences were assigned to viral lineages according to the nomenclature proposed in Rambaut et al. (2020), using the Pangolin web application^10^. Subsequently, an international dataset composed by all genomes available at GISAID^11^ as of July 31 was screened for sequences harboring the B.1.1.33 characteristic mutations ORF6:T27299C and/or N:T29148C. As a reference dataset, we also download from GISAID high quality (<10% of ambiguous positions) complete (>29 kilobases) genome sequences belonging to the global lineage B.1.1 (*n* = 7,766), which also prevails in Brazil. To reduce the size of the global B.1.1 dataset while preserving the full genetic diversity, we construct a “non-redundant” subset (*n* = 3,053) by grouping sequences by similarity (sequence identity cut-off = 1.0) with the CD-HIT program (Li, 2018) and then selecting one representative sequence from each group. SARS-CoV-2 B.1.1 complete genome sequences were aligned using MAFFT v7.467 (Katoh and Standley, 2013) and subject to maximum likelihood (ML) phylogenetic analysis. The ML phylogenetic tree was inferred using IQ-TREE v1.6.12 (Nguyen et al., 2015) under the GTR + F + I + G4 nucleotide substitution model, as selected by the ModelFinder application (Kalyaanamoorthy et al., 2017), and the branch support was assessed by the approximate likelihood-ratio test based on the Shimodaira–Hasegawa-like procedure (SH-aLRT) with 1,000 replicates. The ML phylogenetic tree was visualized using the software FigTree v1.4^12^.

### Analysis of Temporal Signal and Phylogeographic Structure

All genomes identified as B.1.1.33 and closely related basal sequences were submitted to the Bayesian phylogenetic analysis in MrBayes program (Ronquist et al., 2012), under the GTR + I + G substitution model. Two Markov Chain Monte Carlo (MCMC) were run for 10 million generations and stationarity (constant mean and variance of trace plots) and good mixing (effective sample size > 200) were assessed using TRACER v1.7 (Rambaut et al., 2018). The temporal signal was assessed from the maximum clade credibility (MCC) tree by performing a regression analysis of the root-to-tip divergence against sampling time using TempEst (Rambaut et al., 2016). The degree of phylogeographic structure (phylogenetic clustering by sampling location) was investigated using the BaTS program (Parker et al., 2008), which assesses phylogeny-trait association in a posterior sampling of trees. This analysis included metrics as association index (AI), the parsimony score (PS), and the maximum clade (MC) and compared to a null hypothesis generated by tip randomization. Results were considered significant for *P* < 0.01.

### Bayesian Phylogeographic Analyses

The age of the most recent common ancestor (*T*_*MRCA*_) and the spatial diffusion pattern of the B.1.1.33 lineage and closely related basal sequences were jointly estimated using a Bayesian MCMC approach implemented in BEAST 1.10 (Suchard et al., 2018), using the BEAGLE library v3 (Ayres et al., 2019) to improve computational time. Time-scaled Bayesian trees were estimated under GTR + I + G nucleotide substitution model and a strict molecular clock with a uniform substitution rate prior (8–10 × 10^–4^ substitutions/site/year) based on previous estimates (Duchene et al., 2020; Ghafari et al., 2020). The non-parametric Bayesian skyline (BSKL) (Drummond et al., 2005) and Bayesian skygrid (BSKG) (Gill et al., 2013) coalescent models were applied to adjust the demographic signal contained in the dataset. Viral migrations across time were reconstructed using a reversible discrete phylogeographic model (Lemey et al., 2009) with a CTMC rate reference prior (Ferreira and Suchard, 2008). For each analysis, two MCMC chains were run for 100–250 million generations and then combined. Stationarity and good mixing for all parameter estimates were assessed using TRACER v1.7 (Rambaut et al., 2018) as explained above. The maximum clade credibility (MCC) tree was summarized with TreeAnnotator v1.10 and visualized using the FigTree v1.4 program.

### Effective Reproductive Number (R_*e*_) Estimation

To estimate the *R*_*e*_ of the B.1.1.33 lineage in Rio de Janeiro state through time, we used the birth-death skyline (BDSKY) model (Stadler et al., 2013) implemented within BEAST2 v2.6.2 (Bouckaert et al., 2019). The sampling rate (δ) was set to zero for the period prior to the oldest sample and estimated from the data afterward. The BDSKY prior settings for the model parameters are shown in Supplementary Table 2. Origin parameter was conditioned to the root height, and the *R*_*e*_ was estimated in a piece-wise manner over seven time-intervals defined from the date of the most recent sample (April 30, 2020) up to the root of the tree. One MCMC chain was run for 50 million generations and then checked for stationarity and mixing as explained above.

## Results

### Prevalence of SARS-CoV-2 Lineage B.1.1.33 in Brazil and Worldwide

**FIGURE 1:**
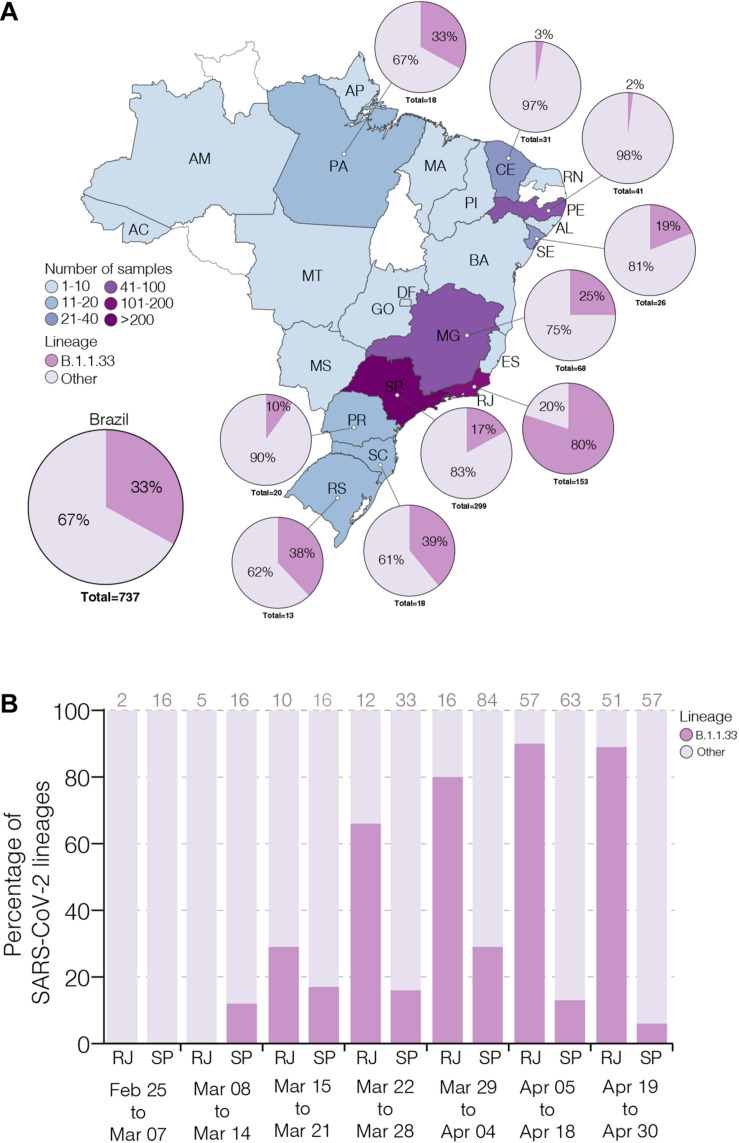
Prevalence of severe acute respiratory syndrome coronavirus-2 (SARS-CoV-2) B.1.1.33 lineage in Brazil. **(A)** Map showing the prevalence of SARS-CoV-2 B.1.1.33 across Brazilian states with more than 10 viral genomes available. The color of each state represents the number of sequences used in this study. The pie charts show the proportion of sequences that belong to the B.1.1.33 lineage in each Brazilian state with >10 sequences and in the whole country. **(B)** Dynamics of B.1.1.33 lineage prevalence in Rio de Janeiro (RJ) and Sao Paulo (SP) states over time. The total of sequences analyzed in each period is indicated above each column. The two letter codes defining the Brazilian states follow the ISO 3166-2:BR standard.

To estimate the prevalence of lineage B.1.1.33 across different Brazilian states over the first 2 months of the epidemic, we generated 190 high-quality viral whole-genomes (>99% coverage) from individuals with confirmed SARS-CoV-2 infection between February 29 and April 30, 2020 that reside in 13 different Brazilian states from the Southeast (*n* = 94, 49%), Northeast (*n* = 66, 35%), North (*n* = 14, 7%), South (*n* = 11, 6%), and Central-West (*n* = 5, 3%) regions (Supplementary Table 1). These SARS-CoV-2 Brazilian sequences here obtained (*n* = 190, 26%) were then combined with Brazilian sequences available in GISAID on July 31 (*n* = 737, 100%) (Supplementary Table 3). The overall prevalence of the lineage B.1.1.33 among Brazilian samples was 33% (*n* = 244/737), but with high variability across regions and time. The highest prevalence of this lineage was observed in the Southeastern state of Rio de Janeiro (80%, *n* = 122/153), while the lowest prevalence was observed in the Northeastern states of Ceara (3%, *n* = 1/31) and Pernambuco (2%, *n* = 1/41) (Figure 1A). Longitudinal analyses in the most heavily affected states, Rio de Janeiro and Sao Paulo, also revealed very different temporal dynamics of lineage B.1.1.33 prevalence (Figure 1B). In Rio de Janeiro, there was a fast increase in the relative proportion of B.1.1.33 up to 90% (*n* = 110/122) in early April and a sustained high prevalence during the following weeks. While in Sao Paulo, there was a more gradual increase in prevalence of lineage B.1.1.33 up to 30% (*n* = 90/299) in early April and a subsequent decline toward the end of the month. None of the 23 early SARS-CoV-2 Brazilian sequences sampled between February 25 and March 7, 2020, mostly derived from imported cases, belong to lineage B.1.1.33. The lineage B.1.1.33 was first detected in Brazil on March 9 in Sao Paulo state and between March 12 and 19 in different states from all country regions (Supplementary Table 3).

To estimate the prevalence of lineage B.1.1.33 outside Brazil, we analyzed almost 75,000 SARS-CoV-2 complete genome sequences sampled worldwide available in GISAID on July 31. We estimated a moderate prevalence (5–18%) of lineage B.1.1.33 across countries from the South American cone (Argentina, Chile and Uruguay), and a very low prevalence (<1%) in some countries from North America (Canada and United States), Europe (England, Portugal, and Scotland), and Oceania (Australia) (Supplementary Table 3). This finding could not be explained by sampling bias as North America, Europe, and Oceania comprise the most densely sampled countries worldwide and clearly suggests that lineage B.1.1.33 was much prevalent in Brazil and other countries from the Southern cone than in any other geographic region. One interesting finding was the detection of some B.1.1.33 strains outside Brazil at very early times, including one virus sampled in Argentina on March 1 (the oldest B.1.1.33 virus detected so far) and viruses sampled in the United States and Canada on March 7, in England on March 12, in Chile and Australia on March 17, and in Portugal on March 18. Thus, the lineage B.1.1.33 was first detected in Argentina, Canada, and the United States at the first week of March 2020, in Brazil and England at the second week of March, and in Australia, Chile, and Portugal at the third week of March. The nearly simultaneous detection of lineage B.1.1.33 in different countries from the Americas and Europe as well as in different Brazilian regions is consistent with a period of cryptic circulation and dissemination of this lineage before its detection in early March 2020.

### Origin and Worldwide Dissemination of SARS-CoV-2 Lineages B.1.1.33

**FIGURE 2:**
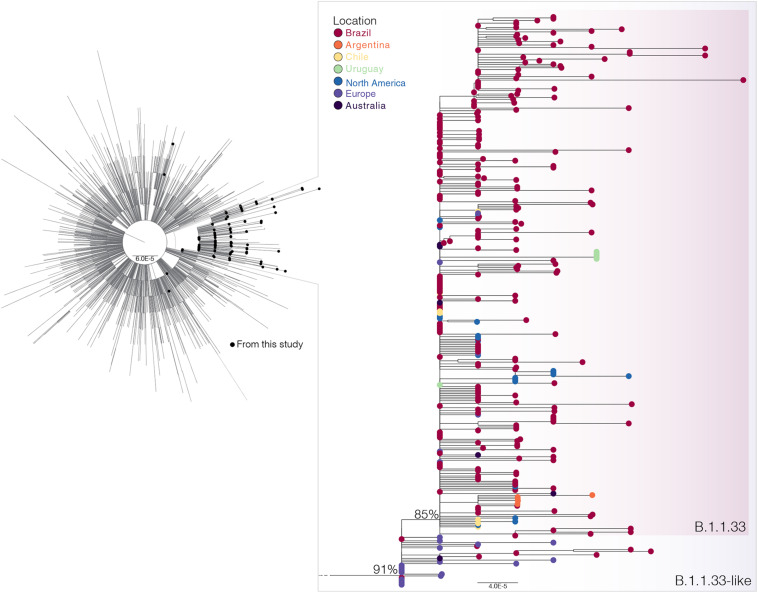
Phylogenetic relationships of SARS-CoV-2 B.1.1 Brazilian and global strains. Maximum likelihood (ML) phylogenetic tree of 190 B.1.1 Brazilian genomes obtained in this survey (black circles) along with 3,053 B.1.1 international reference sequences from the GISAID database. The zoomed view shows the clusters B.1.1.33-like and B.1.1.33. Tip circles are colored according to the sampling location. Only node supports (aLRT) above 70% are shown. Shaded boxes highlight the position of clusters B.1.1.33-like and B.1.1.33. Tree was rooted on midpoint, and branch lengths are drawn to scale with the bars at the bottom indicating nucleotide substitutions per site.

To investigate in more detail the origin of the lineage B.1.1.33, we thoroughly searched all B.1.1 sequences available in GISAID on July 31 (*n* = 7,766) for the presence of the B.1.1.33 lineage-defining non-synonymous synapomorphies (ORF6:T27299C and/or N:T29148C). That search retrieved all B.1.1.33 sequences and a group of 28 B.1.1 sequences (19 from Europe, eight from Brazil, and one from Australia) that only harbor the mutation N:T29148C (Supplementary Table 4). These sequences, here named B.1.1.33-like, were aligned with all B.1.1.33 sequences described above and with a reference dataset of 3,053 B.1.1 sequences representing the global diversity of that lineage. The ML phylogenetic analysis revealed that the B.1.1.33 and B.1.1.33-like sequences branched together in a highly supported (SH-*aLRT* = 91%) monophyletic clade, with the B.1.1.33 sequences grouping in a monophyletic subcluster (SH-*aLRT* = 85%) nested among the basal B.1.1.33-like sequences (Figure 2). Most (85%, *n* = 19/22) B.1.1.33-like sequences from Europe were sampled between February 2 and April 6, 2020, and all Brazilian B.1.1.33-like sequences were sampled between March 13 and 19 in the states of Minas Gerais and Federal District. The overall prevalence of lineage B.1.1.33-like was low in Brazil (1.1%, *n* = 8/737) and outside (<1%, *n* = 20/3,926) but was moderately high (10%, *n* = 7/68) in the Brazilian state of Minas Gerais (Supplementary Table 4). None of the individuals from Minas Gerais infected with the B.1.1.33-like viruses reported international travel, supporting that they correspond to local transmission (Xavier et al., 2020). Interestingly, the earliest B.1.1.33-like strain detected in Brazil was identical to some of those detected in Europe (Supplementary Table 5).

**FIGURE 3:**
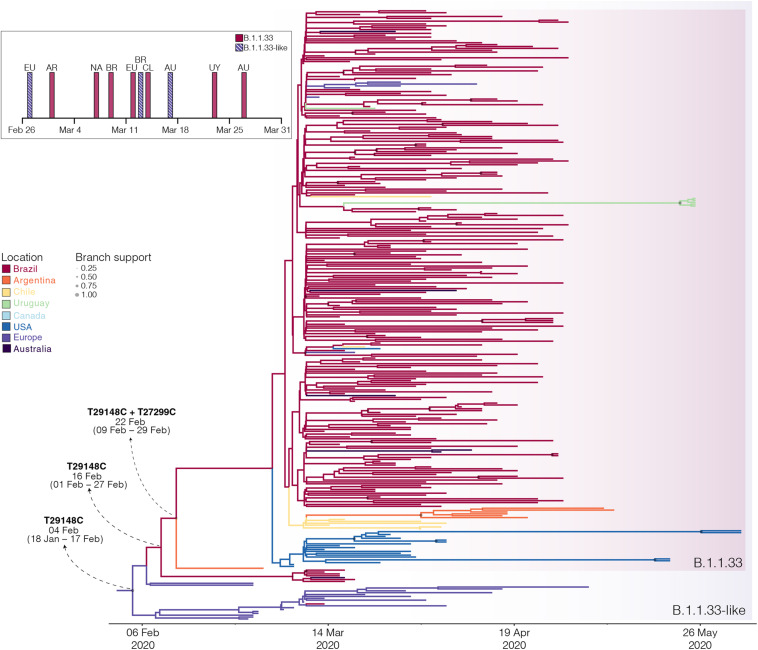
Spatiotemporal dissemination of the SARS-CoV-2 clades B.1.1.33-like and B.1.1.33. Time-scaled Bayesian phylogeographic MCC tree of the major B.1.1 lineages circulating in Brazil. Branches are colored according to the most probable location state of their descendent nodes as indicated at the legend. Circle sizes at internal nodes are proportional to the corresponding posterior probability support as indicated at the legend. The inferred T_*MRCA*_ (based on the median of the posterior heights) and nucleotide substitutions fixed at ancestral key nodes are shown. Shaded boxes highlight the position of the clades B.1.1.33-like and B.1.1.33. The tree is automatically rooted under the assumption of a strict molecular clock, and all horizontal branch lengths are drawn to a scale of years. The inset figure depicts the timeline of the earliest detection of clades B.1.1.33-like (blue dashed bars) and B.1.1.33 (red bars) in Europe (EU), North America (NA), Australia (AU), Argentina (AR), Brazil (BR), Chile (CL), and Uruguay (UY).

**FIGURE 4:**
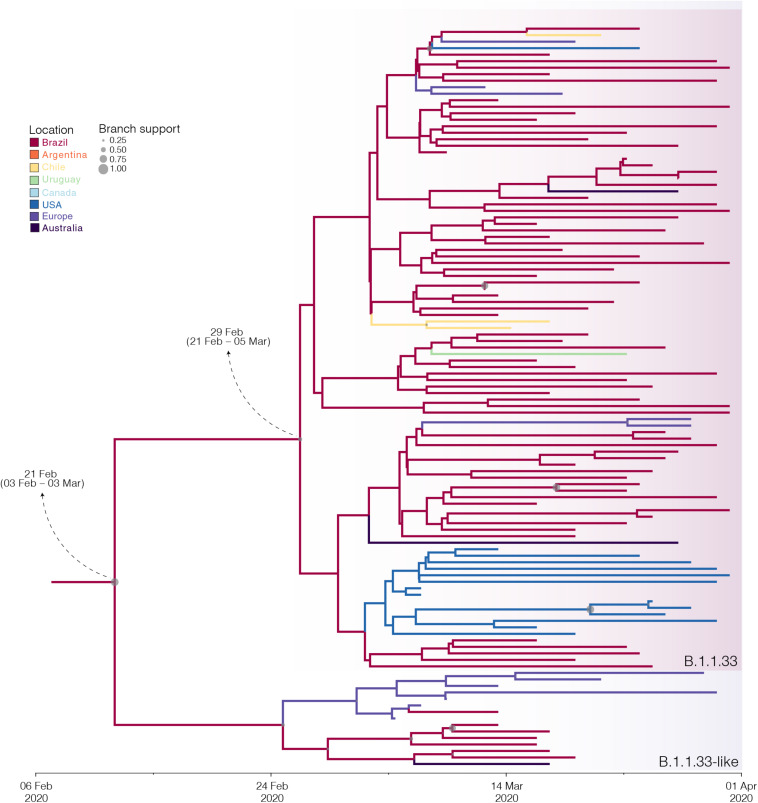
Influence of sampling on the phylogeographic reconstruction of SARS-CoV-2 clade B.1.1.33. Time-scaled Bayesian phylogeographic maximum clade credibility (MCC) tree of a subset of sequences comprising the oldest B.1.1.33-like and B.1.1.33 strains. Branches are colored according to the most probable location state of their descendent nodes as indicated at the legend. Circle size at internal nodes are proportional to the corresponding posterior probability support as indicated at the legend. The inferred T_*MRCA*_ (based on the median of the posterior heights) and nucleotide substitutions fixed at ancestral key nodes are shown. Shaded boxes highlight the position of the clades B.1.1.33-like and B.1.1.33. The tree is automatically rooted under the assumption of a strict molecular clock, and all horizontal branch lengths are drawn to a scale of years.

The B.1.1.33 and B.1.1.33-like sequences sampled worldwide were next combined and analyzed using Bayesian phylogeographic approaches. The worldwide B.1.1.33/B.1.1.33-like dataset displayed a significant geographic structure (Supplementary Table 6), but a weak temporal structure (*R*^2^ = 0.07) (Supplementary Figure 1) and time-scaled Bayesian trees were thus reconstructed using a stringent molecular clock prior. The BSKG analysis traced the origin of the B.1.1.33-like lineage most probably to Europe [Posterior state probability (PSP) = 0.89] on February 4 [95% high posterior density (HPD): January 18 to February 17], its dissemination to Brazil around February 16 (95% HPD: February 1–27), and the origin of lineage B.1.1.33 to Brazil (*PSP* = 0.86) on February 22 (95% HPD: February 9–29) (Figure 3). The paucity of SARS-CoV-2 Brazilian sequences sampled in February and early March (Figure 3, inset), however, might represent a critical constraint for accurate phylogeographic reconstructions. To test this hypothesis, we conducted a new phylogeographic analysis with a subset of 16 B.1.1.33-like sequences (eight from Brazil, seven from Europe, and one from Australia) and 98 B.1.1.33 sequences (72 from Brazil) sampled between March 5 and 31. The new sampling scheme had a tremendous impact on the inferred transmission history as it traced the most probable origin of both B.1.1.33-like (*PSP* = 0.93) and B.1.1.33 (*PSP* = 0.99) lineages to Brazil on February 21 (95% HPD: February 3 to March 3) and February 29 (95% HPD: February 21 to March 5), respectively (Figure 4 and Supplementary Table 7). Thus, any conclusion about the spatial and temporal origin of lineages B.1.1.33-like and B.1.1.33 should be interpreted with caution.

### Dissemination Dynamics of SARS-CoV-2 Lineage B.1.1.33 in Rio de Janeiro

**FIGURE 5:**
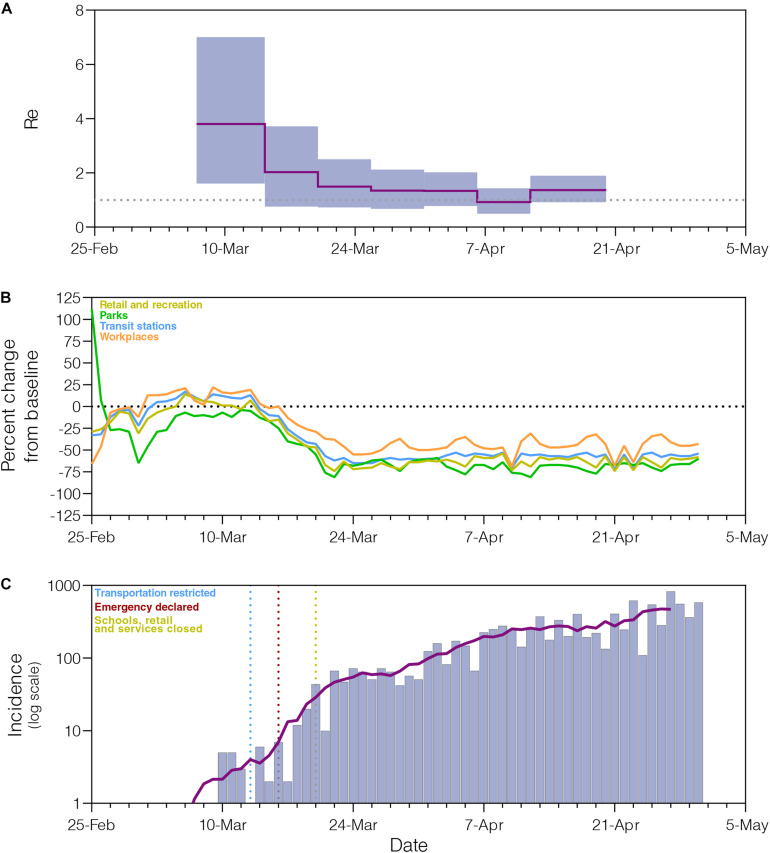
Epidemiological and mobility indicators of the SARS-CoV-2 epidemic in Rio de Janeiro. **(A)** Temporal variation of the effective reproductive number (Re) of the B.1.1.33 lineage in Rio de Janeiro estimated using the Bayesian birth–death approach. **(B)** Mobility data trends reported as percentage change measured against baseline. Each parameter is colored following the legend. **(C)** Progress of incidence of SARS-CoV-2 in Rio de Janeiro. The line represents the weekly average. The date of the main epidemic control measures is indicated by the vertical dotted lines.

The phylogeographic structure of the B.1.1.33 lineage circulating in different Brazilian states did not differ from the null hypotheses of panmixis (Supplementary Table 8), demonstrating that the low genetic diversity and rapid spread of the B.1.1.33 lineage in Brazil imposes an important constraint to reconstruct the early within-country geographic dispersion pattern of this viral lineage. Phylogenomic modeling of the B.1.1.33 lineage, however, could offer important clues about the epidemic dynamics and effectiveness of control measures in those Brazilian states where the COVID-19 epidemic was dominated by this viral variant, like the Rio de Janeiro state. Indeed, the analysis of B.1.1.33 sequences from Rio de Janeiro using the BDSKY model (Figure 5A) supports an epidemic pattern that is fully consistent with changes in human mobility according to the Google Community Mobility Reports (Figure 5B) and with temporal trend of new diagnoses of SARS-CoV-2 (Figure 5C). The BDSKY model suggests a steep (66%) decrease in the *R*_*e*_ from 3.8 (95% HPD: 1.6–7.0) in late February and beginning in March, to 1.3 (95% HPD: 0.7–2.1) by the end of March, which coincides with the implementation of different control measures in this Brazilian state between March 10 and 20 that drastically reduce both human mobility, and the rate of incidence increase. Our phylogenomic analysis also suggests that the median *Re* stabilizes just above 1.0 during April, with the exception of a short interval between the 6th and 12th when median *Re* was about one, consistent with a transient stabilization of incidence trend during the second week of April. Thus, although early social distancing measures were able to drastically decrease the *Re* of the lineage B.1.1.33 in the Rio de Janeiro state, they probably failed to bring its spread under control (*Re* < 1).

## Discussion

Our genomic survey confirms that SARS-CoV-2 lineage B.1.1.33 is responsible for a substantial fraction of the early community viral transmissions in Brazil (∼33%, *n* = 244/737), but it is not homogenously distributed across the country (Candido et al., 2020). The estimated prevalence of lineage B.1.1.33 ranges from 2% (*n* = 1/41) of the SARS-CoV-2 genomes sampled in Pernambuco state to 80% (*n* = 122/153) in Rio de Janeiro between February and April 2020. Our study also suggests that lineage B.1.1.33 followed different temporal trends across Brazilian states. The prevalence of lineage B.1.1.33 rapidly increased during March in Rio de Janeiro, reaching up to 90% (*n* = 110/122) of SARS-CoV-2 genomes sampled by early April. In Sao Paulo, by contrast, this lineage displayed a more gradual increase in prevalence up to 30% (*n* = 90/299) of viral genomes sampled in early April and then declined toward the end of the month. These findings support that SARS-CoV-2 genetic diversity exhibits great variation across different Brazilian states and that in-depth molecular epidemiologic studies at a local scale should be necessary to fully understand the complex dynamics of the COVID-19 epidemic in different locations in the country during the early phase.

Our study shows for the first time that lineage B.1.1.33 was not unique to Brazil, but also circulated in other countries from the Americas, Europe, and Australia. The lineage B.1.1.33 exhibits a medium prevalence (5–20%) in South American countries from the Southern Cone and a very low prevalence (<1%) in countries from North America, Europe, and Oceania. Notably, we found that lineage B.1.1.33 strain was first detected in Argentina (March 1) and North America (March 7) before Brazil (Sao Paulo state, March 9). This may reflect the origin and earliest circulation of lineage B.1.1.33 outside Brazil or may be the consequence of limitations of public health systems to detect the early introduction and local circulation of SARS-CoV-2. Indeed, most SARS-CoV-2 cases detected in the very early phase of Brazilian epidemic (<March 5, 2020) correspond to imported cases, and community viral transmission was only officially recognized on March 20 (Melo et al., 2020). The paucity of diagnosis of local SARS-CoV-2 cases in Brazil during February and early March certainly might have limited the ability to detect early circulation of the autochthonous SARS-CoV-2 Brazilian lineage B.1.1.33.

The lineage B.1.1.33 was originally defined by the presence of two non-synonymous synapomorphies in ORF6 (T27299C/I33T) and the nucleocapsid protein (T29148C/I292T) (Candido et al., 2020). Our analysis of over 7,000 B.1.1 sequences available on GISAID initiative, however, revealed a group of 28 sequences (19 from Europe, eight from Brazil, and one from Australia) that also harbor mutation N:T29148C. These sequences, designated here as B.1.1.33-like, branched basal to lineage B.1.1.33 and represents an evolutionary intermediate between lineages B.1.1 and B.1.1.33. Thus, mutation N:T29148C is a synapomorphy common to lineages B.1.1.33-like and B.1.1.33. Lineage B.1.1.33-like was first detected in Netherlands and Switzerland (February 2–29) and only later in Brazil (March 13–19, 2020) and displayed a very low prevalence in Europe (<1%) and Brazil (1%). Although this viral lineage was only detected in a few early samples from Brazil, epidemiological data supports local transmission of this viral lineage in the Southeastern state of Minas Gerais (Xavier et al., 2020). It is currently unclear whether the lineage B.1.1.33-like represents an extinct viral variant or continues to be transmitted among Brazilian and/or European populations.

Several lines of evidence support that clades B.1.1.33 and B.1.1.33-like circulated for several weeks before being detected in symptomatic carriers in Brazil between March 9 and 13, 2020. Our phylogeographic reconstructions with the complete dataset traced the introduction of lineage B.1.1.33-like to Brazil on February 16 (95% HPD: February 1 to February 27) and the subsequent local origin of lineage B.1.1.33 on February 22 (95% HPD: February 9–29), consistent with a previous estimate (Candido et al., 2020). The nearly simultaneous detection of clade B.1.1.33 in distant states from all Brazilian regions between March 9 and 19 is also compatible with a period of untracked local circulation of this variant. Our findings are also supported by epidemiological surveillance data (available from: https://covid.saude.gov.br/) that reveals the onset symptoms of the first SARS-CoV-2-positive cases with severe acute respiratory illness in Brazil since February 16–22. Also, in agreement with our results, a recent study based on projections from the cumulative number of deaths during the early phase estimated the establishment of local transmission of SARS-CoV-2 in Brazil between early and mid-February 2020 (Delatorre et al., 2020).

**FIGURE 6:**
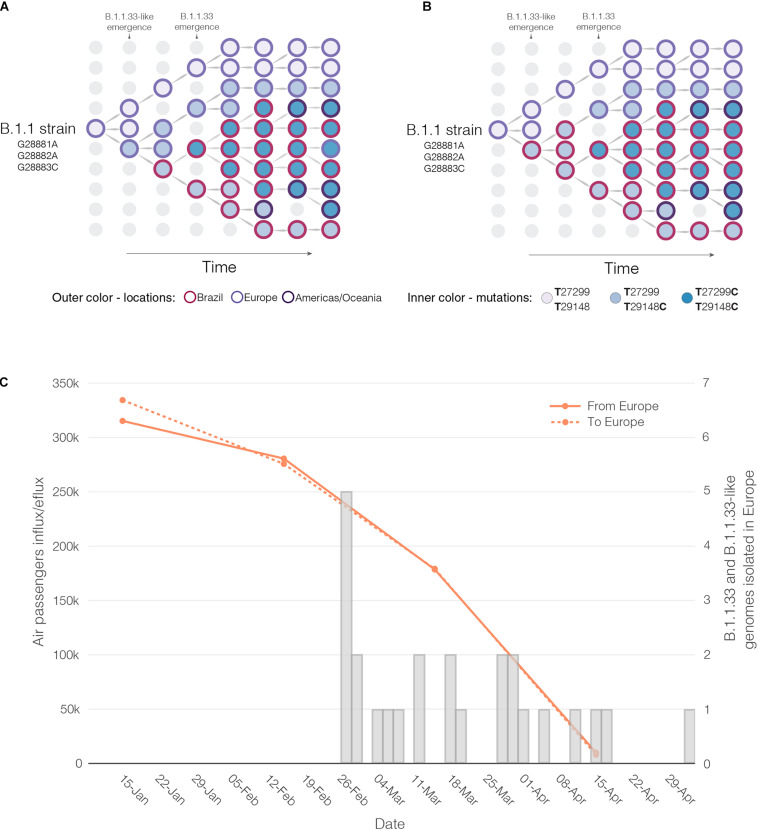
Putative origin and transmission history of the SARS-CoV-2 clades B.1.1.33-like and B.1.1.33. **(A)** Diagrams showing two alternative scenarios for the origin and dissemination of clades B.1.1.33-like and B.1.1.33. The left panel depicts the hypothetical scenario where a B.1.1.33-like strain carrying the mutation T29148C was introduced into Brazil from Europe (marked with an asterisk) and after a period of local transmission in Brazil arose the B.1.1.33 variant carrying the mutation T27299C, which dispersed all over the country and from Brazil to other countries in the Americas and Oceania. **(B)** This panel depicts the hypothetical scenario where a B.1.1 strain was introduced from Europe to Brazil, and mutations T29148C and T27299C arose at sequential steps during local transmission. According to this second scenario, Brazil was the epicenter of dissemination of both clades B.1.1.33-like and B.1.1.33 to other countries in Europe, the Americas, and Oceania. **(C)** Graphic showing the monthly number of international air passengers from South America, North America, and Europe that arrived in Brazil during 2020 (available at: https://www.anac.gov.br) (left-hand axis) along with probability density of T_*MRCA*_ estimates for clades B.1.1.33.like (gray) and B.1.1.33 (red).

Reconstructions of the spatial and temporal origin of SARS-CoV-2 lineages B.1.1.33-like and B.1.1.33 could be biased due the uneven spatial and temporal sampling during early phase as was described for other lineages (Lemey et al., 2020; Villabona-Arenas et al., 2020; Worobey et al., 2020). Indeed, our phylogeographic analyses supports two different viral transmission histories according to the dataset analyzed. Analysis of the complete dataset supports that lineage B.1.1.33-like most probably arose in Europe and was later disseminated to Brazil, where it spread and gave origin to lineage B.1.1.33 (Figure 6A). When early B.1.1.33-like sequences sampled in Europe at late February were removed, however, phylogeographic analysis indicates that both lineages originated at sequential steps during viral local spread in Brazil (Figure 6B). This second phylogenetic hypothesis is consistent with some epidemiological data. First, the extremely low prevalence of the clade B.1.1.33-like in Europe makes its transmission to Brazil a highly unlikely epidemiological scenario. Second, the paucity of B.1.1.33-like and B.1.1.33 sequences detected in Europe after early April 2020 coincides with a sharp decrease in the flow of international air travels to/from Brazil (Figure 6C), suggesting that those infections might be associated to imported cases from Brazil.

Our analyses support that lineage B.1.1.33 was exported from Brazil to neighboring South American countries (Argentina, Chile, and Uruguay) and also to more distant countries (i.e., Canada, United States, Portugal, United Kingdom, and Australia) at multiple times. It is interesting to note that most (>80%, *n* = 40/53) B.1.1.33 sequences detected in Europe, North America, Australia, and Chile were sampled before implementation of international air travel restrictions to/from Brazil around early April. By contrast, nearly all B.1.1.33 sequences detected in South American countries of Argentina and Uruguay were sampled between mid-April and late May, thus long after Brazil closed its land borders and limited international air travels. These findings support that air travel restrictions were able to limit the long-distance dispersion of lineage B.1.1.33 from Brazil, but closing of land borders were not so efficient to limit the short-distance dissemination to neighboring South American countries. Our phylogeographic reconstruction also suggests that lineage B.1.1.33 might have seeded secondary outbreaks in Argentina and Uruguay, but those findings should be interpreted with caution because of the relative low number of B.1.1.33 sequences available from those countries.

Our study shows a random phylogenetic clustering of B.1.1.33 strains from most Brazilian states, supporting the notion that SARS-CoV-2 transmission clusters do not contain sufficient phylogenetic information to allow reliable within-country phylogeographic inferences (Mavian et al., 2020; Villabona-Arenas et al., 2020). Despite this limitation, our results highlight the potential of genomic data to inform about the disease dynamics and the effect of public health interventions in specific geographic settings in Brazil. The fast-increasing prevalence of lineage B.1.1.33 in Rio de Janeiro supports that this clade was a major driver of community transmission in this Brazilian state. Phylodynamic modeling indicates a significant reduction of 66% in the median *R*_*e*_ (from 3.8 to 1.3) of lineage B.1.1.33 following non-pharmaceutical interventions in Rio de Janeiro, but also points that such interventions probably failed to bring *R*_*e*_ below 1.0 during the early phase. These findings are fully consistent with the pattern inferred from epidemiological modeling in Rio de Janeiro (Candido et al., 2020; de Souza et al., 2020; Mellan et al., 2020) and contrast with data from Europe and Oceania that points to stronger reductions in *R*_*e*_ (71–82%) after public health interventions, sufficient to bring the epidemic under control (Binny et al., 2020; Flaxman et al., 2020; Jarvis et al., 2020; Seemann et al., 2020).

In summary, this study confirms that SARS-CoV-2 B.1.1.33 is a widespread lineage associated with community transmission in Brazil but also reveals a high geographic compartmentalization of SARS-CoV-2 genetic diversity across states. While lineage B.1.1.33 was a major driver of community transmission in Rio de Janeiro, it displayed a very low prevalence in other heavily affected Brazilian sates. Our study demonstrates that Brazilian lineage B.1.1.33 probably evolved from a B.1.1.33-like basal lineage that also carries the synapomorphic mutation at the nucleocapsid protein. Both viral clades probably circulated in Brazil since the early or mid-February 2020 and reached all Brazilian regions and other countries around the world by mid-March 2020. Finally, our data supports that public health interventions at the early epidemic phase were successful in slowing down the transmission of B.1.1.33 lineage in Rio de Janeiro state, but failed to fully control its dissemination, which broadly reflects the past and ongoing COVID-19 dynamics in the state.

## Data Availability Statement

The datasets presented in this study can be found in online repositories. The names of the repository/repositories and accession number(s) can be found below: GISAID (https://www.gisaid.org/), accession numbers in Supplementary Table 1.

## Ethics Statement

The studies involving human participants were reviewed and approved by FIOCRUZ-IOC Ethics Committee (68118417.6.0000.5248 and CAAE 32333120.4.0000.5190) and the Brazilian Ministry of Health SISGEN (A1767C3). Written informed consent to participate in this study was provided by the participants’ legal guardian/next of kin.

## Author Contributions

PR, ED, TG, DM, FM, GB, and MS conceived, designed the analysis, and wrote the manuscript. PR, ED, TG, DM, FM, LA, AP, AM, MO, BC, GW, CD, MS, JA, ES, SS, SF, LV, LC, JF, VN, CS, IR, MC, and GB collected the data and performed experiments. PR, ED, TG, DM, FM, and GB conceived, designed, and performed the analysis. All authors reviewed and agreed with the content of the manuscript.

## Conflict of Interest

The authors declare that the research was conducted in the absence of any commercial or financial relationships that could be construed as a potential conflict of interest.

## Footnotes

1 https://www.gisaid.org/

2 https://nextstrain.org/ncov/global?c=region

3 https://pangolin.cog-uk.io/

4 https://github.com/articnetwork/rampart

5 https://digitalinsights.qiagen.com

6 https://artic.network/ncov-2019/ncov2019-bioinformatics-sop.html

7 https://github.com/rrwick/Porechop

8 https://github.com/nanoporetech/medaka

9 https://github.com/artic-network/artic-ncov2019

10 https://pangolin.cog-uk.io

11 https://www.gisaid.org/

12 http://tree.bio.ed.ac.uk/software/figtree/
