# Supplementary figures and images for: Evolutionary Dynamics and Dissemination Pattern of the SARS-CoV-2 Lineage B.1.1.33 During the Early Pandemic Phase in Brazil
Source: Front Microbiol. 2021 Feb 17;11:615280. doi: 10.3389/fmicb.2020.615280 (PMC7925893; doi:10.3389/fmicb.2020.615280)

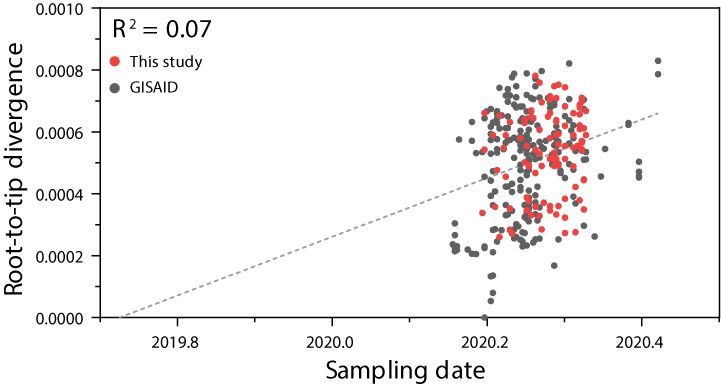

Supplement: Supplementary file 1 [file Image_1.TIF]
