# Supplementary material for: Evolutionary Dynamics and Dissemination Pattern of the SARS-CoV-2 Lineage B.1.1.33 During the Early Pandemic Phase in Brazil
Source: Front Microbiol. 2021 Feb 17;11:615280. doi: 10.3389/fmicb.2020.615280 (PMC7925893; doi:10.3389/fmicb.2020.615280)
Supplement: Supplementary file 2 [file Data_Sheet_1.docx]

Supplementary Material

**Supplementary Figure 1.** Linear regression analysis between the sampling date of each viral sequence and the root-to-tip divergence (genetic distance of that sequence to the tree root) of a ML phylogeny of the SARS-CoV-2 lineages B.1.1.33 and B.1.1.33-like. SARS-CoV-2 genomes generated in this study (red) were combined with other genomes available on the GISAID database (gray).

**Supplementary Figure 1.** Linear regression analysis between the sampling date of each viral sequence and the root-to-tip divergence (genetic distance of that sequence to the tree root) of a ML phylogeny of the SARS-CoV-2 clades B.1.1.EU/BR and B.1.1.BR. SARS-CoV-2 genomes generated in this study (red) were combined with other genomes available on the GISAID database (gray).

| Virus name | Accession number GISAID | Clinical Specimen | Pangolin lineage* | State/Town | Collection date | International travel history | Gender | Age | Clinical outcome |
| --- | --- | --- | --- | --- | --- | --- | --- | --- | --- |
| hCoV-19/Brazil/PR-5617/2020 | EPI_ISL_541340 | NPS | B.1 | Parana / Cianorte | 17/03/2020 | no | Female | 41y | unknown |
| hCoV-19/Brazil/PR-5621/2020 | EPI_ISL_541344 | NPS | B.1.1.28 | Parana / Vere | 19/03/2020 | Cruise ship in Brazilian Coast | Male | 37y | unknown |
| hCoV-19/Brazil/PR-5619/2020 | EPI_ISL_541342 | NPS | B.1 | Parana / Ponta Grossa | 16/03/2020 | Dubai | Female | 61y | unknown |
| hCoV-19/Brazil/PR-5620/2020 | EPI_ISL_541343 | NPS | B.1.1.28 | Parana / Maringa | 20/03/2020 | Emirados Arabes | Male | 63y | unknown |
| hCoV-19/Brazil/SC-769/2020 | EPI_ISL_427306 | NPS | B.1.1 | Santa Catarina / Florianopolis | 10/03/2020 | Europe | Female | 59y | outpatient |
| hCoV-19/Brazil/RJ-477/2020 | EPI_ISL_427300 | NPS | B.1.1 | Rio de Janeiro / Rio de Janeiro | 11/03/2020 | Europe | Male | 48y | outpatient |
| hCoV-19/Brazil/RJ-477i/2020 | EPI_ISL_427301 | Vero E6 | B.1.1 | Rio de Janeiro / Rio de Janeiro | 11/03/2020 | Europe | Male | 48y | outpatient |
| hCoV-19/Brazil/SC-766/2020 | EPI_ISL_427305 | NPS | B.6 | Santa Catarina / Joinvile | 10/03/2020 | Europe and Africa | Male | 57y | unknown |
| hCoV-19/Brazil/PR-5622/2020 | EPI_ISL_541345 | NPS | B.1 | Parana / Curitiba | 28/03/2020 | France | Female | 23y | unknown |
| hCoV-19/Brazil/ES-225/2020 | EPI_ISL_415128 | NPS | B.2.1 | Espirito Santo / Vila Velha | 29/02/2020 | Italy | Female | 37y | inpatient |
| hCoV-19/Brazil/BA-312/2020 | EPI_ISL_415105 | NPS | B.1.1 | Bahia / Feira de Santana | 04/03/2020 | Italy | Female | 34y | inpatient |
| hCoV-19/Brazil/RJ-314/2020 | EPI_ISL_414045 | NPS | B.1 | Rio de Janeiro / Rio de Janeiro | 04/03/2020 | Italy | Female | 52y | outpatient |
| hCoV-19/Brazil/SC-771/2020 | EPI_ISL_541371 | NPS | B.1 | Santa Catarina / Balneario Camburiu | 13/03/2020 | Italy | Female | 52y | unknown |
| hCoV-19/Brazil/PR-5618/2020 | EPI_ISL_541341 | NPS | B.1 | Parana / Curitiba | 17/03/2020 | Italy. | Male | 59y | unknown |
| hCoV-19/Brazil/PR-5623/2020 | EPI_ISL_541346 | NPS | B.1.3 | Parana / Medianeira | 26/03/2020 | Mexico | Male | 27y | unknown |
| hCoV-19/Brazil/SC-770/2020 | EPI_ISL_541370 | NPS | B.1.1.33 | Santa Catarina / Braco do Norte | 12/03/2020 | no | Male | 52y | unknown |
| hCoV-19/Brazil/AP-161167-IEC/2020 | EPI_ISL_450873 | Sputum | B.1.1.33 | Amapa / Macapa | 17/03/2020 | no | Female | 36y | unknown |
| hCoV-19/Brazil/AC-162535-IEC/2020 | EPI_ISL_458139 | NPS | B.1.1.33 | Acre / Rio Branco | 18/03/2020 | no | Male | 81y | unknown |
| hCoV-19/Brazil/SE-6536/2020 | EPI_ISL_541376 | NPS | B.1.1.33 | Sergipe / Nossa Senhora da Gloria | 19/03/2020 | no | Female | 71y | outpatient |
| hCoV-19/Brazil/RJ-763/2020 | EPI_ISL_427302 | NPS | B.1.1.33 | Rio de Janeiro / Petropolis | 20/03/2020 | no | Female | 47y | outpatient |
| hCoV-19/Brazil/RJ-818/2020 | EPI_ISL_427303 | NPS | B.1.1.33 | Rio de Janeiro / Rio de Janeiro | 25/03/2020 | no | Female | 54y | outpatient |
| hCoV-19/Brazil/RJ-899/2020 | EPI_ISL_456071 | NPS | B.1.1 | Rio de Janeiro / Rio de Janeiro | 30/03/2020 | no | Male | 42y | outpatient |
| hCoV-19/Brazil/RJ-1056/2020 | EPI_ISL_456072 | NPS | B.1.1.33 | Rio de Janeiro / Rio de Janeiro | 01/04/2020 | no | Female | 31y | outpatient |
| hCoV-19/Brazil/RJ-1058/2020 | EPI_ISL_456073 | NPS | B.1.1.33 | Rio de Janeiro / Rio de Janeiro | 01/04/2020 | no | Male | 27y | outpatient |
| hCoV-19/Brazil/RJ-1065/2020 | EPI_ISL_456074 | NPS | B.1.1.33 | Rio de Janeiro / Rio de Janeiro | 01/04/2020 | no | Female | 85y | outpatient |
| hCoV-19/Brazil/RJ-1402/2020 | EPI_ISL_456079 | NPS | B.1.1.33 | Rio de Janeiro / Rio de Janeiro | 03/04/2020 | no | Female | 55y | outpatient |
| hCoV-19/Brazil/AP-162966-IEC/2020 | EPI_ISL_458142 | NPS | B.1.1.33 | Amapa / Macapa | 05/04/2020 | no | Female | 37y | unknown |
| hCoV-19/Brazil/RJ-1464/2020 | EPI_ISL_456080 | NPS | B.1.1.33 | Rio de Janeiro / Petropolis | 06/04/2020 | no | Male | 30y | outpatient |
| hCoV-19/Brazil/RJ-1466/2020 | EPI_ISL_456081 | NPS | B.1.1.33 | Rio de Janeiro / Rio de Janeiro | 06/04/2020 | no | Female | 54y | outpatient |
| hCoV-19/Brazil/PA-162802-IEC/2020 | EPI_ISL_458140 | NPS | B.1.1 | Para / Belem | 07/04/2020 | no | Male | 63y | inpatient |
| hCoV-19/Brazil/RJ-1701/2020 | EPI_ISL_456086 | NPS | B.1.1.33 | Rio de Janeiro / Rio de Janeiro | 08/04/2020 | no | Male | 60y | outpatient |
| hCoV-19/Brazil/RJ-1702/2020 | EPI_ISL_456087 | NPS | B.1.1.33 | Rio de Janeiro / Rio de Janeiro | 08/04/2020 | no | Male | 40y | outpatient |
| hCoV-19/Brazil/RJ-1902/2020 | EPI_ISL_456090 | NPS | B.1.1.33 | Rio de Janeiro / Rio de Janeiro | 09/04/2020 | no | Female | 39y | outpatient |
| hCoV-19/Brazil/RJ-1921/2020 | EPI_ISL_456091 | NPS | B.1.1.33 | Rio de Janeiro / Rio de Janeiro | 09/04/2020 | no | Female | 70y | outpatient |
| hCoV-19/Brazil/RJ-1923/2020 | EPI_ISL_456092 | NPS | B.1.1.33 | Rio de Janeiro / Rio de Janeiro | 10/04/2020 | no | Male | 5m | unknown |
| hCoV-19/Brazil/RJ-1927/2020 | EPI_ISL_456093 | NPS | B.1.1.33 | Rio de Janeiro / Rio de Janeiro | 12/04/2020 | no | Male | 46y | outpatient |
| hCoV-19/Brazil/RJ-1948/2020 | EPI_ISL_456095 | NPS | B.1.1.33 | Rio de Janeiro / Rio de Janeiro | 13/04/2020 | no | Female | 45y | inpatient |
| hCoV-19/Brazil/RJ-1952/2020 | EPI_ISL_456096 | NPS | B.1.1.33 | Rio de Janeiro / Rio de Janeiro | 13/04/2020 | no | Male | 29y | outpatient |
| hCoV-19/Brazil/RJ-1953/2020 | EPI_ISL_541352 | NPS | B.1.1.33 | Rio de Janeiro / Rio de Janeiro | 13/04/2020 | no | Female | 41y | Inpatient |
| hCoV-19/Brazil/RJ-2057/2020 | EPI_ISL_456102 | NPS | B.1.1.33 | Rio de Janeiro / Rio de Janeiro | 16/04/2020 | no | Male | 29y | outpatient |
| hCoV-19/Brazil/PA-164239-IEC/2020 | EPI_ISL_458141 | NPS | B.1.1 | Para / Ananindeua | 26/04/2020 | no | Male | 38y | outpatient |
| hCoV-19/Brazil/SE-6549/2020 | EPI_ISL_541379 | NPS | B.1 | Sergipe / Aracaju | 26/04/2020 | no | Male | 46y | outpatient |
| hCoV-19/Brazil/SE-6563/2020 | EPI_ISL_541385 | NPS | B.1.1.33 | Sergipe / Aracaju | 26/04/2020 | no | Male | 49y | outpatient |
| hCoV-19/Brazil/PA-164684-IEC/2020 | EPI_ISL_458148 | NPS | B.1.1.33 | Para / Belem | 27/04/2020 | no | Male | 38y | unknown |
| hCoV-19/Brazil/SE-6557/2020 | EPI_ISL_541383 | NPS | B.1 | Sergipe / Laranjeiras | 27/04/2020 | no | Male | 37y | outpatient |
| hCoV-19/Brazil/SE-6606/2020 | EPI_ISL_541394 | NPS | B.1 | Sergipe / Aracaju | 20/04/2020 | no | Male | 40y | unknown |
| hCoV-19/Brazil/PA-161548-IEC/2020 | EPI_ISL_450874 | NPS | B.1.1.33 | Para / Maraba | 20/03/2020 | no | Female | 28y | unknown |
| hCoV-19/Brazil/SE-6535/2020 | EPI_ISL_541375 | NPS | B.1.5 | Sergipe / Aracaju | 12/03/2020 | Spain | Female | 36y | outpatient |
| hCoV-19/Brazil/RJ-352/2020 | EPI_ISL_427299 | NPS | A.2 | Rio de Janeiro / Niteroi | 05/03/2020 | unknown | Male | 27y | unknown |
| hCoV-19/Brazil/BA-510/2020 | EPI_ISL_427293 | NPS | B.1.1 | Bahia / Feira de Santana | 06/03/2020 | unknown | Female | 41y | unknown |
| hCoV-19/Brazil/DF-615i/2020 | EPI_ISL_427294 | Vero E6 | B.1.1.33 | Federal District / Brasilia | 13/03/2020 | unknown | Male | unknown | unknown |
| hCoV-19/Brazil/DF-619i/2020 | EPI_ISL_427295 | Vero E6 | B.1.1.33 | Federal District / Brasilia | 13/03/2020 | unknown | Male | unknown | unknown |
| hCoV-19/Brazil/AL-837/2020 | EPI_ISL_427292 | NPS | B.1.1 | Alagoas / Maceio | 18/03/2020 | unknown | Male | 65y | unknown |
| hCoV-19/Brazil/SE-6533/2020 | EPI_ISL_541374 | NPS | B.1.1 | Sergipe / Aracaju | 19/03/2020 | unknown | Female | 55y | outpatient |
| hCoV-19/Brazil/SE-6530/2020 | EPI_ISL_541373 | NPS | B.1.1 | Sergipe / Aracaju | 20/03/2020 | unknown | Female | 20y | unknown |
| hCoV-19/Brazil/DF-891/2020 | EPI_ISL_427298 | NPS | B.1.1.33 | Federal District / Brasilia | 22/03/2020 | unknown | Female | 61y | Deceased |
| hCoV-19/Brazil/DF-861/2020 | EPI_ISL_427296 | NPS | B.1.1.33 | Federal District / Brasilia | 23/03/2020 | unknown | Male | 55y | unknown |
| hCoV-19/Brazil/DF-862/2020 | EPI_ISL_427297 | NPS | B.1.1.33 | Federal District / Brasilia | 23/03/2020 | unknown | Female | 25y | unknown |
| hCoV-19/Brazil/RJ-1111/2020 | EPI_ISL_456076 | NPS | B.1.1.33 | Rio de Janeiro / Rio de Janeiro | 25/03/2020 | unknown | Male | 59y | unknown |
| hCoV-19/Brazil/RJ-872/2020 | EPI_ISL_427304 | NPS | B.1.1 | Rio de Janeiro / Rio de Janeiro | 26/03/2020 | unknown | Female | 60y | unknown |
| hCoV-19/Brazil/SE-6529/2020 | EPI_ISL_541372 | NPS | B.1.1.28 | Sergipe / Aracaju | 27/03/2020 | unknown | Female | 61y | inpatient |
| hCoV-19/Brazil/RJ-1100/2020 | EPI_ISL_456075 | NPS | B.1.1.33 | Rio de Janeiro / Belford Roxo | 02/04/2020 | unknown | Male | 30y | outpatient |
| hCoV-19/Brazil/RJ-1555/2020 | EPI_ISL_467344 | NPS | B.2.2 | Rio de Janeiro / Petropolis | 02/04/2020 | unknown | Male | 31y | unknown |
| hCoV-19/Brazil/RJ-1574/2020 | EPI_ISL_467345 | NPS | B.1.1.33 | Rio de Janeiro / Rio de Janeiro | 02/04/2020 | unknown | Male | 82y | unknown |
| hCoV-19/Brazil/RJ-1595/2020 | EPI_ISL_467346 | NPS | B.2.2 | Rio de Janeiro / Rio de Janeiro | 02/04/2020 | unknown | Female | 83y | unknown |
| hCoV-19/Brazil/RJ-1600/2020 | EPI_ISL_456082 | NPS | B.1.1.33 | Rio de Janeiro / Teresopolis | 02/04/2020 | unknown | Male | 68y | unknown |
| hCoV-19/Brazil/AP-162741-IEC/2020 | EPI_ISL_458138 | Sputum | B.1.1.33 | Amapa / Macapa | 03/04/2020 | unknown | Female | 37y | unknown |
| hCoV-19/Brazil/RJ-1627/2020 | EPI_ISL_456083 | NPS | B.1.1.33 | Rio de Janeiro / Nova Iguacu | 03/04/2020 | unknown | Female | 44y | unknown |
| hCoV-19/Brazil/RJ-1783/2020 | EPI_ISL_541347 | NPS | B.1.1.33 | Rio de Janeiro / Rio de Janeiro | 04/04/2020 | unknown | Female | 50y | unknown |
| hCoV-19/Brazil/RJ-1830/2020 | EPI_ISL_541349 | NPS | B.1.1.33 | Rio de Janeiro / Sao Goncalo | 04/04/2020 | unknown | Female | 48y | unknown |
| hCoV-19/Brazil/RJ-1862/2020 | EPI_ISL_541350 | NPS | B.1.1.33 | Rio de Janeiro / Rio de Janeiro | 04/04/2020 | unknown | Female | 72y | unknown |
| hCoV-19/Brazil/RJ-1870/2020 | EPI_ISL_541351 | NPS | B.1 | Rio de Janeiro / Rio de Janeiro | 05/04/2020 | unknown | Female | 85y | unknown |
| hCoV-19/Brazil/MA-163069-IEC/2020 | EPI_ISL_458149 | NPS | B.1.1.33 | Maranhao / Sao Luis | 06/04/2020 | unknown | Female | 40y | unknown |
| hCoV-19/Brazil/PE-IAM215/2020 | EPI_ISL_500471 | NPS | B.1.1 | Pernambuco / Olinda | 06/04/2020 | unknown | unknown | unknown | unknown |
| hCoV-19/Brazil/RJ-1719/2020 | EPI_ISL_456088 | NPS | B.1.1 | Rio de Janeiro / Itaborai | 06/04/2020 | unknown | Female | 36y | unknown |
| hCoV-19/Brazil/RJ-1798/2020 | EPI_ISL_541348 | NPS | B.1.1.33 | Rio de Janeiro / Mesquita | 06/04/2020 | unknown | Male | 19y | unknown |
| hCoV-19/Brazil/PE-IAM08/2020 | EPI_ISL_500460 | NPS | B.1.1 | Pernambuco / Recife | 07/04/2020 | unknown | Female | 44y | Recovered |
| hCoV-19/Brazil/PE-IAM10/2020 | EPI_ISL_500461 | NPS | B.1.1 | Pernambuco / Recife | 07/04/2020 | unknown | Female | 55y | Recovered |
| hCoV-19/Brazil/PE-IAM16/2020 | EPI_ISL_500865 | NPS | B.1.1 | Pernambuco / Recife | 07/04/2020 | unknown | Male | 33y | Recovered |
| hCoV-19/Brazil/PE-IAM17/2020 | EPI_ISL_500866 | NPS | B.1.1 | Pernambuco / Recife | 07/04/2020 | unknown | Female | 36y | Recovered |
| hCoV-19/Brazil/PE-IAM18/2020 | EPI_ISL_500868 | NPS | B.1.1 | Pernambuco / Recife | 07/04/2020 | unknown | Male | 27y | Recovered |
| hCoV-19/Brazil/PE-IAM19/2020 | EPI_ISL_500467 | NPS | B.1 | Pernambuco / Recife | 07/04/2020 | unknown | Female | 28y | Recovered |
| hCoV-19/Brazil/PE-IAM226/2020 | EPI_ISL_500474 | NPS | B.1.1 | Pernambuco / Jaboatao dos Guararapes | 07/04/2020 | unknown | Male | 56y | Recovered |
| hCoV-19/Brazil/PE-IAM89/2020 | EPI_ISL_500486 | NPS | B.1.1 | Pernambuco / Camaragibe | 07/04/2020 | unknown | Male | 81y | Recovered |
| hCoV-19/Brazil/RJ-2155/2020 | EPI_ISL_541354 | NPS | B.1.1.28 | Rio de Janeiro / Niteroi | 07/04/2020 | unknown | Female | 43y | unknown |
| hCoV-19/Brazil/PE-IAM221/2020 | EPI_ISL_500473 | NPS | B.1.1 | Pernambuco / Recife | 08/04/2020 | unknown | Female | 44y | Recovered |
| hCoV-19/Brazil/PE-IAM273/2020 | EPI_ISL_500476 | NPS | B.1.1 | Pernambuco / Recife | 08/04/2020 | unknown | Male | 80y | Deceased |
| hCoV-19/Brazil/PE-IAM29/2020 | EPI_ISL_500872 | NPS | B.1.1 | Pernambuco / Olinda | 08/04/2020 | unknown | Male | 86y | Deceased |
| hCoV-19/Brazil/PE-IAM30/2020 | EPI_ISL_500477 | NPS | B.1.1 | Pernambuco / Ipojuca | 08/04/2020 | unknown | Male | 31y | Recovered |
| hCoV-19/Brazil/PE-IAM39/2020 | EPI_ISL_500481 | NPS | B.1.1 | Pernambuco / Recife | 08/04/2020 | unknown | Female | 48y | Recovered |
| hCoV-19/Brazil/PE-IAM48/2020 | EPI_ISL_500482 | NPS | B.1.1 | Pernambuco / Recife | 08/04/2020 | unknown | Male | 67y | Recovered |
| hCoV-19/Brazil/PE-IAM67/2020 | EPI_ISL_500483 | NPS | B.1.1.28 | Pernambuco / Recife | 08/04/2020 | unknown | Male | 30y | Recovered |
| hCoV-19/Brazil/PE-IAM84/2020 | EPI_ISL_500484 | NPS | B.1.1 | Pernambuco / Recife | 08/04/2020 | unknown | Male | 20y | Recovered |
| hCoV-19/Brazil/PE-IAM87/2020 | EPI_ISL_500485 | NPS | B.1.1 | Pernambuco / Sao Lourenco da Mata | 08/04/2020 | unknown | Male | 28y | Recovered |
| hCoV-19/Brazil/RJ-1690/2020 | EPI_ISL_456084 | NPS | B.1.1.33 | Rio de Janeiro / Rio de Janeiro | 08/04/2020 | unknown | Female | 42y | outpatient |
| hCoV-19/Brazil/RJ-1691/2020 | EPI_ISL_456085 | NPS | B.1.1.33 | Rio de Janeiro / Rio de Janeiro | 08/04/2020 | unknown | Male | 42y | outpatient |
| hCoV-19/Brazil/PE-IAM103/2020 | EPI_ISL_500462 | NPS | B.1.1 | Pernambuco / Recife | 09/04/2020 | unknown | Male | 36y | Recovered |
| hCoV-19/Brazil/PE-IAM138/2020 | EPI_ISL_500463 | NPS | B.1 | Pernambuco / Recife | 09/04/2020 | unknown | Female | 37y | Recovered |
| hCoV-19/Brazil/PE-IAM139/2020 | EPI_ISL_500464 | NPS | B.1.1 | Pernambuco / Recife | 09/04/2020 | unknown | Female | 30y | Recovered |
| hCoV-19/Brazil/PE-IAM158/2020 | EPI_ISL_500465 | NPS | B.1.1 | Pernambuco / Recife | 09/04/2020 | unknown | Female | 33y | Recovered |
| hCoV-19/Brazil/PE-IAM167/2020 | EPI_ISL_500466 | NPS | B.1.1 | Pernambuco / Recife | 09/04/2020 | unknown | Female | 38y | Recovered |
| hCoV-19/Brazil/PE-IAM171/2020 | EPI_ISL_500867 | NPS | B.1.1 | Pernambuco / Recife | 09/04/2020 | unknown | Female | 54y | Recovered |
| hCoV-19/Brazil/PE-IAM209/2020 | EPI_ISL_500468 | NPS | B.1.1 | Pernambuco / Recife | 09/04/2020 | unknown | Male | 44y | Recovered |
| hCoV-19/Brazil/PE-IAM211/2020 | EPI_ISL_500469 | NPS | B.1.1 | Pernambuco / Recife | 09/04/2020 | unknown | unknown | unknown | unknown |
| hCoV-19/Brazil/PE-IAM212/2020 | EPI_ISL_500470 | NPS | B.1.1 | Pernambuco / Recife | 09/04/2020 | unknown | unknown | unknown | unknown |
| hCoV-19/Brazil/PE-IAM220/2020 | EPI_ISL_500472 | NPS | B.1.1 | Pernambuco / Recife | 09/04/2020 | unknown | Male | 56y | Recovered |
| hCoV-19/Brazil/PE-IAM230/2020 | EPI_ISL_500869 | NPS | B.1.1 | Pernambuco / Recife | 09/04/2020 | unknown | Female | 51y | Recovered |
| hCoV-19/Brazil/PE-IAM235/2020 | EPI_ISL_500870 | NPS | B.1.1 | Pernambuco / Recife | 09/04/2020 | unknown | Male | 66y | Recovered |
| hCoV-19/Brazil/PE-IAM238/2020 | EPI_ISL_500475 | NPS | B.1.1 | Pernambuco / Recife | 09/04/2020 | unknown | Female | 52y | Recovered |
| hCoV-19/Brazil/PE-IAM248/2020 | EPI_ISL_500871 | NPS | B.1.1 | Pernambuco / Recife | 09/04/2020 | unknown | Female | 42y | Recovered |
| hCoV-19/Brazil/RJ-1901/2020 | EPI_ISL_456089 | NPS | B.1.1.33 | Rio de Janeiro / Rio de Janeiro | 09/04/2020 | unknown | Female | 59y | outpatient |
| hCoV-19/Brazil/PE-IAM291/2020 | EPI_ISL_500873 | NPS | B.1.1 | Pernambuco / Recife | 13/04/2020 | unknown | Female | 44y | Recovered |
| hCoV-19/Brazil/PE-IAM305/2020 | EPI_ISL_500478 | NPS | B.1.1 | Pernambuco / Camaragibe | 13/04/2020 | unknown | Female | 23y | Recovered |
| hCoV-19/Brazil/PE-IAM307/2020 | EPI_ISL_500479 | NPS | B.1.1.33 | Pernambuco / Alianca | 13/04/2020 | unknown | unknown | unknown | unknown |
| hCoV-19/Brazil/PE-IAM311/2020 | EPI_ISL_500480 | NPS | B.1.1 | Pernambuco / Recife | 13/04/2020 | unknown | Female | 48y | Recovered |
| hCoV-19/Brazil/PE-IAM355/2020 | EPI_ISL_500874 | NPS | B.1.1 | Pernambuco / Olinda | 13/04/2020 | unknown | Female | 42y | Recovered |
| hCoV-19/Brazil/PE-IAM356/2020 | EPI_ISL_500875 | NPS | B.1.1.28 | Pernambuco / Paulista | 13/04/2020 | unknown | Female | 42y | Recovered |
| hCoV-19/Brazil/RJ-1943/2020 | EPI_ISL_456094 | NPS | B.1.1.33 | Rio de Janeiro / Rio de Janeiro | 13/04/2020 | unknown | Female | 38y | outpatient |
| hCoV-19/Brazil/RJ-1966/2020 | EPI_ISL_456097 | NPS | B.1.1.33 | Rio de Janeiro / Rio de Janeiro | 13/04/2020 | unknown | Male | unknown | outpatient |
| hCoV-19/Brazil/RJ-2000/2020 | EPI_ISL_456098 | NPS | B.1.1.33 | Rio de Janeiro / Rio de Janeiro | 13/04/2020 | unknown | Female | 29y | unknown |
| hCoV-19/Brazil/RJ-2007/2020 | EPI_ISL_456099 | NPS | B.1.1.33 | Rio de Janeiro / Rio de Janeiro | 13/04/2020 | unknown | Female | 58y | unknown |
| hCoV-19/Brazil/RJ-2192/2020 | EPI_ISL_541355 | NPS | B.1.1.28 | Rio de Janeiro / Rio de Janeiro | 13/04/2020 | unknown | Female | 59y | unknown |
| hCoV-19/Brazil/AP-163972-IEC/2020 | EPI_ISL_458144 | Sputum | B.1.1.33 | Amapa / Macapa | 15/04/2020 | unknown | Male | 44y | unknown |
| hCoV-19/Brazil/AP-164082-IEC/2020 | EPI_ISL_458143 | Sputum | B.1.1.33 | Amapa / Macapa | 15/04/2020 | unknown | Male | 44y | unknown |
| hCoV-19/Brazil/RJ-2033/2020 | EPI_ISL_456100 | NPS | B.1.1.33 | Rio de Janeiro / Rio de Janeiro | 15/04/2020 | unknown | Female | 37y | unknown |
| hCoV-19/Brazil/RJ-2044/2020 | EPI_ISL_456101 | NPS | B.1.1.33 | Rio de Janeiro / Duque de Caxias | 15/04/2020 | unknown | Female | 28y | unknown |
| hCoV-19/Brazil/RJ-2062/2020 | EPI_ISL_456103 | NPS | B.1.1 | Rio de Janeiro / Rio de Janeiro | 16/04/2020 | unknown | Female | 49y | unknown |
| hCoV-19/Brazil/RJ-2072/2020 | EPI_ISL_456104 | NPS | B.1.1.33 | Rio de Janeiro / Rio de Janeiro | 16/04/2020 | unknown | Female | 51y | unknown |
| hCoV-19/Brazil/RJ-2077/2020 | EPI_ISL_456105 | NPS | B.1.1.33 | Rio de Janeiro / Rio de Janeiro | 16/04/2020 | unknown | Female | 38y | unknown |
| hCoV-19/Brazil/RJ-2078/2020 | EPI_ISL_456106 | NPS | B.1.1.33 | Rio de Janeiro / Rio de Janeiro | 16/04/2020 | unknown | Female | 34y | unknown |
| hCoV-19/Brazil/RJ-2091/2020 | EPI_ISL_467347 | NPS | B.1.1.33 | Rio de Janeiro / Rio de Janeiro | 16/04/2020 | unknown | Female | 51y | unknown |
| hCoV-19/Brazil/RJ-2100/2020 | EPI_ISL_541353 | NPS | B.1.1.33 | Rio de Janeiro / Rio de Janeiro | 16/04/2020 | unknown | Male | 41y | unknown |
| hCoV-19/Brazil/RJ-2195/2020 | EPI_ISL_467348 | NPS | B.1.1.33 | Rio de Janeiro / Rio de Janeiro | 17/04/2020 | unknown | Male | 45y | unknown |
| hCoV-19/Brazil/RJ-2197/2020 | EPI_ISL_467349 | NPS | B.1.1.33 | Rio de Janeiro / Rio de Janeiro | 17/04/2020 | unknown | Male | 32y | unknown |
| hCoV-19/Brazil/RJ-2208/2020 | EPI_ISL_467350 | NPS | B.1.1.33 | Rio de Janeiro / Rio de Janeiro | 17/04/2020 | unknown | Male | 43y | unknown |
| hCoV-19/Brazil/RJ-2231/2020 | EPI_ISL_541356 | NPS | B.1.1.33 | Rio de Janeiro / Rio de Janeiro | 17/04/2020 | unknown | Male | 34y | unknown |
| hCoV-19/Brazil/RJ-2233/2020 | EPI_ISL_467351 | NPS | B.1.1.33 | Rio de Janeiro / Rio de Janeiro | 17/04/2020 | unknown | Female | 30y | unknown |
| hCoV-19/Brazil/RJ-2342/2020 | EPI_ISL_541357 | NPS | B.1.1.33 | Rio de Janeiro / not informed | 20/04/2020 | unknown | Female | 64y | unknown |
| hCoV-19/Brazil/RJ-2343/2020 | EPI_ISL_541358 | NPS | B.1.1.33 | Rio de Janeiro / not informed | 20/04/2020 | unknown | Male | 20y | unknown |
| hCoV-19/Brazil/RJ-2369/2020 | EPI_ISL_541359 | NPS | B.1.1.28 | Rio de Janeiro / Rio de Janeiro | 20/04/2020 | unknown | Male | 25y | unknown |
| hCoV-19/Brazil/RJ-2376/2020 | EPI_ISL_541360 | NPS | B.1.1.33 | Rio de Janeiro / Niteroi | 20/04/2020 | unknown | Male | 36y | unknown |
| hCoV-19/Brazil/RJ-2377/2020 | EPI_ISL_541361 | NPS | B.1.1.33 | Rio de Janeiro / Rio de Janeiro | 20/04/2020 | unknown | Female | 38y | unknown |
| hCoV-19/Brazil/RJ-2422/2020 | EPI_ISL_467352 | NPS | B.1.1.33 | Rio de Janeiro / Queimados | 20/04/2020 | unknown | Female | 27y | inpatient |
| hCoV-19/Brazil/SE-6579/2020 | EPI_ISL_541388 | NPS | B.1.1.33 | Sergipe / Aracaju | 20/04/2020 | unknown | Female | 49y |  |
| hCoV-19/Brazil/SE-6583/2020 | EPI_ISL_541389 | NPS | B.1 | Sergipe / Aracaju | 20/04/2020 | unknown | Male | 37y | unknown |
| hCoV-19/Brazil/SE-6601/2020 | EPI_ISL_541392 | NPS | B.1.1.33 | Sergipe / Aracaju | 20/04/2020 | unknown | Female | 42y | unknown |
| hCoV-19/Brazil/SE-6607/2020 | EPI_ISL_541395 | NPS | B.1 | Sergipe / Cabedelo | 20/04/2020 | unknown | Male | 37y | unknown |
| hCoV-19/Brazil/SE-6608/2020 | EPI_ISL_541396 | NPS | B.1 | Sergipe / Aracaju | 20/04/2020 | unknown | Male | 41y | unknown |
| hCoV-19/Brazil/SE-6603/2020 | EPI_ISL_541393 | NPS | B.1.1 | Sergipe / Tomar do Geru | 21/04/2020 | unknown | Male | 6y | unknown |
| hCoV-19/Brazil/PA-164173-IEC/2020 | EPI_ISL_458146 | NPS | B.1.1 | Para / Ananindeua | 23/04/2020 | unknown | Female | 38y | unknown |
| hCoV-19/Brazil/PA-164218-IEC/2020 | EPI_ISL_458147 | NPS | B.1.1 | Para / Belem | 24/04/2020 | unknown | Male | 45y | unknown |
| hCoV-19/Brazil/RJ-2669/2020 | EPI_ISL_467353 | NPS | B.1.1.33 | Rio de Janeiro / Niteroi | 24/04/2020 | unknown | Female | 25y | outpatient |
| hCoV-19/Brazil/RJ-2676i/2020 | EPI_ISL_467354 | Vero E6 | B.1.5 | Rio de Janeiro / Rio de Janeiro | 24/04/2020 | unknown | Male | 48y | unknown |
| hCoV-19/Brazil/RJ-2678/2020 | EPI_ISL_467355 | NPS | B.1.1.33 | Rio de Janeiro / Rio de Janeiro | 24/04/2020 | unknown | Male | 29y | unknown |
| hCoV-19/Brazil/RJ-2682/2020 | EPI_ISL_467356 | NPS | B.1.1 | Rio de Janeiro / Rio de Janeiro | 24/04/2020 | unknown | Female | 29y | unknown |
| hCoV-19/Brazil/RJ-2683/2020 | EPI_ISL_467357 | NPS | B.1.1.33 | Rio de Janeiro / Rio de Janeiro | 24/04/2020 | unknown | Female | 31y | unknown |
| hCoV-19/Brazil/RJ-2696/2020 | EPI_ISL_467358 | NPS | B.1.1.33 | Rio de Janeiro / Rio de Janeiro | 24/04/2020 | unknown | Female | 65y | unknown |
| hCoV-19/Brazil/RJ-2717/2020 | EPI_ISL_467359 | NPS | B.1.1 | Rio de Janeiro / Rio de Janeiro | 24/04/2020 | unknown | Female | 36y | unknown |
| hCoV-19/Brazil/SE-6567/2020 | EPI_ISL_541386 | NPS | B.1.1.28 | Sergipe / Aracaju | 24/04/2020 | unknown | Female | 36y | unknown |
| hCoV-19/Brazil/SE-6568/2020 | EPI_ISL_541387 | NPS | B.1 | Sergipe / Jaboatiana | 24/04/2020 | unknown | Female | 35y | unknown |
| hCoV-19/Brazil/RJ-2733/2020 | EPI_ISL_467360 | NPS | B.1.1.33 | Rio de Janeiro / Duque de Caxias | 25/04/2020 | unknown | Female | 49y | unknown |
| hCoV-19/Brazil/RJ-2777/2020 | EPI_ISL_467364 | NPS | B.1.1.33 | Rio de Janeiro / Nova Iguacu | 25/04/2020 | unknown | Female | 34y | unknown |
| hCoV-19/Brazil/SE-6539/2020 | EPI_ISL_541377 | NPS | B.1 | Sergipe / Aracaju | 26/04/2020 | unknown | Female | 37y | unknown |
| hCoV-19/Brazil/SE-6544/2020 | EPI_ISL_541378 | NPS | B.1.1 | Sergipe / Estancia | 26/04/2020 | unknown | Female | 37y | unknown |
| hCoV-19/Brazil/RJ-2769/2020 | EPI_ISL_467361 | NPS | B.1.1.33 | Rio de Janeiro / Rio de Janeiro | 27/04/2020 | unknown | Female | 30y | unknown |
| hCoV-19/Brazil/RJ-2770/2020 | EPI_ISL_467362 | NPS | B.1.1.33 | Rio de Janeiro / Rio de Janeiro | 27/04/2020 | unknown | Female | 30y | unknown |
| hCoV-19/Brazil/RJ-2776/2020 | EPI_ISL_467363 | NPS | B.1.1.33 | Rio de Janeiro / Rio de Janeiro | 27/04/2020 | unknown | Male | 64y | unknown |
| hCoV-19/Brazil/RJ-2811/2020 | EPI_ISL_467365 | NPS | B.1.1.33 | Rio de Janeiro / Rio de Janeiro | 27/04/2020 | unknown | Male | 44y | unknown |
| hCoV-19/Brazil/RJ-2812/2020 | EPI_ISL_467366 | NPS | B.1.1 | Rio de Janeiro / Rio de Janeiro | 27/04/2020 | unknown | Male | 38y | unknown |
| hCoV-19/Brazil/RJ-2822/2020 | EPI_ISL_467367 | NPS | B.1.1.33 | Rio de Janeiro / Niteroi | 27/04/2020 | unknown | Male | 62y | inpatient |
| hCoV-19/Brazil/SE-6550/2020 | EPI_ISL_541380 | NPS | B.1 | Sergipe / Aracaju | 27/04/2020 | unknown | Female | 47y | unknown |
| hCoV-19/Brazil/SE-6555/2020 | EPI_ISL_541381 | NPS | B.1 | Sergipe / Riachuelo | 27/04/2020 | unknown | Male | 44y | unknown |
| hCoV-19/Brazil/SE-6556/2020 | EPI_ISL_541382 | NPS | B.1.1.33 | Sergipe / Itaporanga Dajuda | 27/04/2020 | unknown | Male | 27y | unknown |
| hCoV-19/Brazil/SE-6561/2020 | EPI_ISL_541384 | NPS | B.1.1 | Sergipe / Nossa Senhora do Socorro | 27/04/2020 | unknown | Female | 71y | unknown |
| hCoV-19/Brazil/AP-164346-IEC/2020 | EPI_ISL_458145 | Sputum | B.1.1.33 | Amapa / Macapa | 28/04/2020 | unknown | Female | 62y | unknown |
| hCoV-19/Brazil/RJ-2840/2020 | EPI_ISL_467368 | NPS | B.1.1.33 | Rio de Janeiro / Rio de Janeiro | 28/04/2020 | unknown | Male | 36y | unknown |
| hCoV-19/Brazil/RJ-2844/2020 | EPI_ISL_467369 | NPS | B.1.1.33 | Rio de Janeiro / Rio de Janeiro | 28/04/2020 | unknown | Female | 29y | outpatient |
| hCoV-19/Brazil/RJ-2847/2020 | EPI_ISL_467370 | NPS | B.1.1.33 | Rio de Janeiro / Rio de Janeiro | 28/04/2020 | unknown | Female | 35y | unknown |
| hCoV-19/Brazil/RJ-2868/2020 | EPI_ISL_467371 | NPS | B.1.1.33 | Rio de Janeiro / Rio de Janeiro | 28/04/2020 | unknown | Female | 49y | unknown |
| hCoV-19/Brazil/RJ-2903/2020 | EPI_ISL_541362 | NPS | B.1.1.33 | Rio de Janeiro / Rio de Janeiro | 28/04/2020 | unknown | Male | 37y | unknown |
| hCoV-19/Brazil/RJ-2904/2020 | EPI_ISL_541363 | NPS | B.1.1.33 | Rio de Janeiro / Rio de Janeiro | 28/04/2020 | unknown | Female | 27y | unknown |
| hCoV-19/Brazil/RJ-2938/2020 | EPI_ISL_541364 | NPS | B.1.1.33 | Rio de Janeiro / Rio de Janeiro | 29/04/2020 | unknown | Male | 27y | unknown |
| hCoV-19/Brazil/RJ-2950/2020 | EPI_ISL_541365 | NPS | B.1.1.33 | Rio de Janeiro / Rio de Janeiro | 29/04/2020 | unknown | Male | 33y | unknown |
| hCoV-19/Brazil/RJ-2951/2020 | EPI_ISL_541366 | NPS | B.1.1.33 | Rio de Janeiro / Rio de Janeiro | 29/04/2020 | unknown | Female | 38y | unknown |
| hCoV-19/Brazil/RJ-2966/2020 | EPI_ISL_541367 | NPS | B.1.1.33 | Rio de Janeiro / Rio de Janeiro | 29/04/2020 | unknown | Male | 38y | unknown |
| hCoV-19/Brazil/SE-6591/2020 | EPI_ISL_541390 | NPS | B.1 | Sergipe / Aracaju | 29/04/2020 | unknown | Male | 31y | unknown |
| hCoV-19/Brazil/SE-6594/2020 | EPI_ISL_541391 | NPS | B.1 | Sergipe / Aracaju | 29/04/2020 | unknown | Female | 41y | unknown |
| hCoV-19/Brazil/RJ-2999/2020 | EPI_ISL_541368 | NPS | B.1.1.33 | Rio de Janeiro / Niteroi | 30/04/2020 | unknown | Female | 41y | unknown |
| hCoV-19/Brazil/RJ-3021/2020 | EPI_ISL_541369 | NPS | B.1.1.33 | Rio de Janeiro / Rio de Janeiro | 30/04/2020 | unknown | Male | 34y | unknown |
| hCoV-19/Brazil/RJ-1119/2020 | EPI_ISL_456077 | NPS | B.1.1.33 | Rio de Janeiro / Rio de Janeiro | 25/05/2020 | unknown | Male | 55y | unknown |

**Supplementary Table 2.** Prevalence of SARS-CoV-2 lineage B.1.1.33 across Brazilian states.

| **Region** | **State** | **SARS-CoV-2** | **B.1.1.33** | **First detection** | **Last detection** |
| --- | --- | --- | --- | --- | --- |
| Southeast | RJ | 153 | 122 (80%) | 16 Mar | 30 Apr |
|  | MG | 68 | 17 (25%) | 17 Mar | 17Apr |
|  | SP | 299 | 51 (17%) | 09 Mar | 25 Apr |
|  | ES | 1 | - | - | |
|  | **Total SE** | **521** | **190 (36%)** | **09 Mar** | **30 Apr** |
| Northeast | SE | 26 | 5 (19%) | 19 Mar | 27 Apr |
|  | CE | 31 | 1 (3%) | 28 Mar | |
|  | PE | 41 | 1 (2%) | 13 Apr | |
|  | MA | 7 | 6 | 31 Mar | 06 Apr |
|  | BA | 4 | 1 | 31 Mar | |
|  | PI | 1 | 1 | 19 Mar | |
|  | AL | 1 | - | - | |
|  | RN | 2 | - | - | |
|  | **Total NE** | **113** | **15 (13%)** | **19 Mar** | **13 Apr** |
| North | PA | 18 | 6 (33%) | 20 Mar | 27 Apr |
|  | AP | 6 | 6 | 17 Mar | 20 Apr |
|  | AM | 7 | 2 | 02 Apr | |
|  | AC | 1 | 1 | 18 Mar | |
|  | **Total N** | **32** | **15 (47%)** | **17 Mar** | **27 Apr** |
| South | SC | 18 | 7 (39%) | 12 Mar | 03 Apr |
|  | RS | 13 | 5 (38%) | 16 Mar | 02 Apr |
|  | PR | 20 | 2 (10%) | 16 Mar | 02 Apr |
|  | **Total S** | **51** | **14 (27%)** | **12 Mar** | **03 Apr** |
| Central-West | DF | 7 | 5 | 13 Mar | 23 Mar |
|  | GO | 8 | 2 | 03 Apr | 04 Apr |
|  | MT | 4 | 2 | 30 Mar | 03 Apr |
|  | MS | 1 | 1 | 02 Apr | |
|  | **Total CW** | **20** | **10 (50%)** | **13 Mar** | **04 Apr** |
| **Total BR** | | **737** | **244 (33%)** | **09 Mar** | **30 Apr** |

**Supplementary Table 3.** Prevalence of SARS-CoV-2 lineage B.1.1.33 across countries.

| **Region** | **Country** | **SARS-Cov-2** | **B.1.1.33** | **First detection** | **Last detection** |
| --- | --- | --- | --- | --- | --- |
| South America | Brazil | 737 | 244 (33%) | 09 Mar | 30 Apr |
|  | Argentina | 38 | 7 (18%) | 01 Mar | 09 May |
|  | Chile | 167 | 8 (5%) | 14 Mar | 06 Apr |
|  | Uruguay | 74 | 6 (8%) | 23 Mar | 25 May |
| North America | Canada | 1,055 | 1 (<1%) | 07 Mar | |
|  | US | 15,753 | 20 (<1%) | 07 Mar | 03 Jun |
| Oceania | Australia | 2,665 | 5 (<1%) | 27 Mar | 11 Apr |
| Europe | Portugal | 768 | 3 (<1%) | 18 Mar | 28 Mar |
|  | England | 23,319 | 2 (<1%) | 12 Mar | 19 Mar |
|  | Scotland | 5,125 | 1 (<1%) | 12 Apr | |

**Supplementary Table 4.** Prevalence of SARS-CoV-2 lineage B.1.1.33-like across countries.

| **Country** | **State** | **SARS-Cov-2** | **B.1.1.33-like** | **First detection** | **Last detection** |
| --- | --- | --- | --- | --- | --- |
| Brazil | MG* | 68 | 7 | 13 Mar | 19 Mar |
|  | DF | 7 | 1 | 13 Mar | |
|  | Total | 737 | 8 (1.1%) | 13 Mar | 19 Mar |
| United Kingdom | - | 23,319 | 8 (<1%) | 07 Mar | 17 Apr |
| Netherlands | - | 1,605 | 4 (<1%) | 27 Feb | 29 Feb |
| Switzerland | - | 815 | 4 (<1%) | 28 Feb | 21 Mar |
| Czech Republic | - | 49 | 1 (2.0%) | 05 Mar | |
| Scotland | - | 5,125 | 1 (<1%) | 13 Mar | |
| Germany | - | 348 | 1 (<1%) | 05 Apr | |
| Australia | - | 2,665 | 1 (<1%) | 17 Mar | |

* Sequences CV34 and CV45 harbor the substitution T29148C, but displayed an ambiguous nucleotide at position 27299, and were hence not assigned to any specific lineage.

**Supplementary Table 5** List of SARS-CoV-2 B.1.1.33-like sequences identical to the Brazilian sequence EPI_ISL_426580.

| **Country** | **State** | **Sequence ID** | **GISAID ID** | **Date of sampling** |
| --- | --- | --- | --- | --- |
| Brazil | DF | DFBR-0001 | EPI_ISL_426580 | 13 Mar |
|  | MG | CV47 | EPI_ISL_429700 | 19 Mar |
| Netherlands | - | NoordHolland_10001 | EPI_ISL_454750 | 27 Feb |
|  |  | Helmond_1363548 | EPI_ISL_413574 | 29 Feb |
| Switzerland | - | GE1402 | EPI_ISL_415700 | 28 Feb |
|  |  | AG7120 | EPI_ISL_415457 | 29 Feb |
|  |  | GE1736 | EPI_ISL_429203 | 21 Mar |
| England | - | 201101358 | EPI_ISL_464655 | 07 Mar |
|  |  | 201140286 | EPI_ISL_464719 | 09 Mar |
| Scotland | - | EDB019 | EPI_ISL_425825 | 13 Mar |
| Czech Republic | - | ChVir1912 | EPI_ISL_416743 | 05 Mar |

**Supplementary Table 6.** Phylogeny-trait association tests to assess phylogeographic structure of the global B.1.1.33/B.1.1.33-like dataset.

| **Metric** | **Data** | | | **Null hypothesis** | | | ***p* value** |
| --- | --- | --- | --- | --- | --- | --- | --- |
|  | **Mean** | **Lower 95% CI** | **Upper 95% CI** | **Mean** | **Lower 95% CI** | **Upper 95% CI** |  |
| AI | 5.17 | 3.89 | 6.48 | 11.67 | 10.94 | 12.40 | **<0.001** |
| PS | 40.97 | 39.00 | 42.00 | 71.54 | 70.18 | 72.28 | **<0.001** |
| MC (Brazil/*n*=252) | 16.68 | 10.00 | 28.00 | 10.33 | 9.32 | 11.39 | **<0.001** |
| MC (Europe/*n*=25) | 5.17 | 2.00 | 10.00 | 1.42 | 1.22 | 2.03 | **0.001** |
| MC (USA/*n*=20) | 6.97 | 7.00 | 7.00 | 1.29 | 1.12 | 1.99 | **0.001** |
| MC (Argentina/*n*=7) | 5.99 | 6.00 | 6.00 | 1.03 | 1.00 | 1.08 | **0.001** |
| MC (Uruguay/*n*=6) | 5.00 | 5.00 | 5.00 | 1.03 | 1.00 | 1.04 | **0.001** |
| MC (Chile/*n*=8) | 1.05 | 1.00 | 1.00 | 1.05 | 1.01 | 1.12 | 1.000 |
| MC (Australia/*n*=6) | 1.02 | 1.00 | 1.00 | 1.02 | 1.00 | 1.04 | 1.000 |

AI - Association index; PS - Parsimony score; MC - Monophyletic clade

**Supplementary Table 7.** Bayesian estimates of the time of origin and most probable source location of SARS-CoV-2 lineages B.1.1.33-like and B.1.1.33.

| **Model** | T_MRCA_  B.1.1.33-like | Location  B.1.1.33-like | T_MRCA_  B.1.1.33 | Location  B.1.1.33 |
| --- | --- | --- | --- | --- |
| **BSKL+SMC** | 25 Jan  (27 Dec - 16 Feb) | Europe  *PSP* = 0.95 | 26 Feb  (17 Feb - 01 Mar) | Brazil  *PSP* = 0.93 |
| **BSKL+RMC** | 25 Jan  (24 Dec - 18 Feb) | Europe  *PSP* = 0.93 | 26 Feb  (18 Feb - 01 Mar) | Brazil  *PSP* = 0.93 |
| **BSKG+SMC** | 04 Feb  (18 Jan - 17 Feb) | Europe  *PSP* = 0.89 | 22 Feb  (09 Feb - 29 Feb) | Brazil  *PSP* = 0.86 |
| **BSKG+RMC** | 04 Feb  (18 Jan - 17 Feb) | Europe  *PSP* = 0.87 | 21 Feb  (07 Feb - 29 Feb) | Brazil  *PSP* = 0.85 |

BSKL: Bayesian Skyline coalescent model. BSKG: Bayesian Skygrid coalescent model. SMC: Strict molecular clock model. RMC: Relaxed molecular clock model. *PSP*: Posterior state probability

**Supplementary Table 8.** Phylogeny-trait association tests to assess phylogeographic structure of the Brazilian B.1.1.33 dataset.

| **Metric** | **Data** | | | **Null hypothesis** | | | ***p* value** |
| --- | --- | --- | --- | --- | --- | --- | --- |
|  | **Mean** | **Lower 95% CI** | **Upper 95% CI** | **Mean** | **Lower 95% CI** | **Upper 95% CI** |  |
| AI | 12.00 | 10.12 | 13.82 | 16.44 | 15.62 | 17.25 | **<0.001** |
| PS | 105.45 | 101.00 | 110.00 | 114.15 | 112.01 | 115.87 | **<0.001** |
| MC (RJ/*n*=122) | 5.71 | 5.00 | 8.00 | 4.47 | 4.01 | 5.64 | 0.107 |
| MC (SP/*n*=51) | 2.44 | 2.00 | 4.00 | 2.26 | 2.02 | 2.50 | 0.973 |
| MC (MG/*n*=51) | 3.00 | 3.00 | 3.00 | 1.28 | 1.11 | 1.99 | **0.002** |
| MC (SC/*n*=7) | 1.35 | 1.00 | 2.00 | 1.04 | 1.01 | 1.06 | 1.000 |
| MC (AP/*n*=6) | 1.02 | 1.00 | 1.00 | 1.03 | 1.00 | 1.04 | 1.000 |
| MC (MA/*n*=6) | 1.04 | 1.00 | 1.00 | 1.04 | 1.00 | 1.05 | 1.000 |
| MC (PA/*n*=6) | 2.01 | 2.00 | 2.00 | 1.04 | 1.00 | 1.04 | **0.004** |
| MC (DF/*n*=5) | 1.01 | 1.00 | 1.00 | 1.03 | 1.00 | 1.03 | 1.000 |
| MC (RS/*n*=5) | 1.03 | 1.00 | 1.00 | 1.02 | 1.00 | 1.03 | 1.000 |
| MC (SE/*n*=5) | 1.52 | 1.00 | 2.00 | 1.04 | 1.00 | 1.06 | **0.003** |
| MC (AM/*n*=2) | 2.00 | 2.00 | 2.00 | 1.00 | 1.00 | 1.00 | **0.001** |
| MC (GO/*n*=2) | 1.00 | 1.00 | 1.00 | 1.00 | 1.00 | 1.00 | 1.000 |
| MC (MT/*n*=2) | 1.00 | 1.00 | 1.00 | 1.00 | 1.00 | 1.00 | 1.000 |
| MC (PR/*n*=2) | 1.00 | 1.00 | 1.00 | 1.00 | 1.00 | 1.00 | 1.000 |

AI - Association index; PS - Parsimony score; MC - Monophyletic clade

**Supplementary Table 9.** Values and priors for the parameters of the BDSKL model.

| **Parameter** | **Model** | **Prior (Value)** |
| --- | --- | --- |
| Nucleotide substitution model | GTR + I + G | - |
| Molecular clock model | Strict clock | Uniform distribution  (8-10 x 10^-4^) |
| Death rate | - | Exponential distribution  (mean = 36.5) |
| Sampling start time |  | 16/03/2020 |
| Reproductive number |  | Lognormal distribution  (mean = 0.8, sd = 0.5) |
| Sampling proportion* |  | Uniform distribution  (lower = 0, upper = 0.016) |

* Upper bound estimated as the number of analyzed SARS-CoV-2 sequences divided by the number of confirmed cases in Rio de Janeiro.

**Supplementary Table 10.** GISAID acknowledgment table of Global SARS-CoV-2 lineages B.1.1.
